# Surface-Activated Mechano-Catalysis for Ambient Conversion of Plastic Waste

**DOI:** 10.1021/jacs.4c07157

**Published:** 2024-09-09

**Authors:** Adrian H. Hergesell, Renate J. Baarslag, Claire L. Seitzinger, Raghavendra Meena, Patrick Schara, Željko Tomović, Guanna Li, Bert M. Weckhuysen, Ina Vollmer

**Affiliations:** † Inorganic Chemistry and Catalysis Group, Institute for Sustainable and Circular Chemistry, Utrecht University, Utrecht 3584 CG, The Netherlands; ‡ Biobased Chemistry and Technology, Wageningen University, Wageningen 6708 WG, The Netherlands; § Laboratory of Organic Chemistry, Wageningen University, Wageningen 6708 WE, The Netherlands; ∥ Polymer Performance Materials Group, Department of Chemical Engineering and Chemistry, Eindhoven University of Technology, Eindhoven 5600 MB, The Netherlands

## Abstract

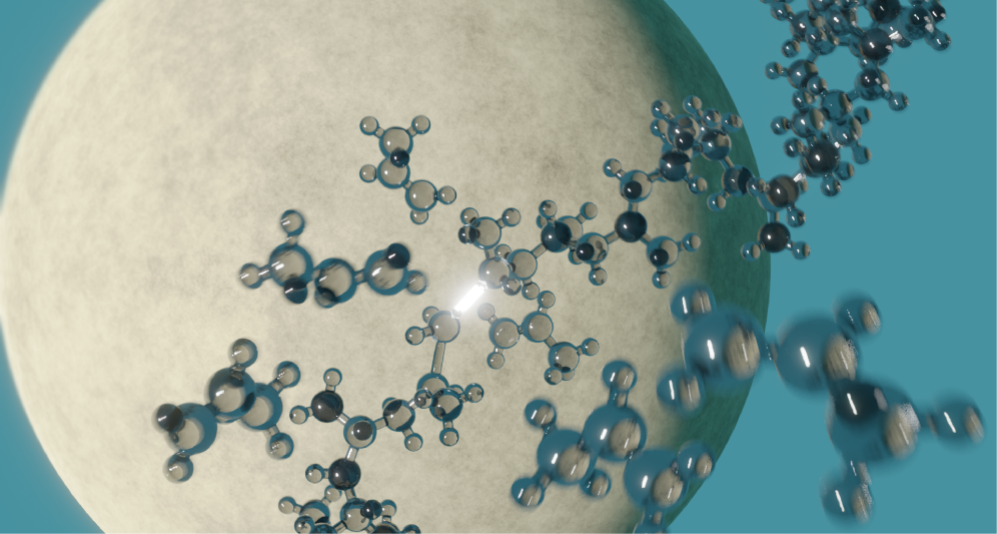

Improved recycling technologies can offer sustainable end-of-life options for plastic waste. While polyolefins can be converted into small hydrocarbons over acid catalysts at high temperatures, we demonstrate an alternative mechano-catalytic strategy at ambient conditions. The mechanism is fundamentally different from classical acidity-driven high-temperature approaches, exploiting mechanochemically generated radical intermediates. Surface activation of zirconia grinding spheres creates redox active surface sites directly at the point of mechanical energy input. This allows control over mechano-radical reactivity, while powder catalysts are not active. Optimized milling parameters enable the formation of 45% C_1–10_ hydrocarbons from polypropylene within 1 h at ambient temperature. While mechanochemical bond scission is undesired in plastic production, we show that it can also be exploited for chemical recycling.

## Introduction

The majority of plastic waste is landfilled, burned, or leaks into the environment.^1^ Waste management and pollution are multifaceted topics to be addressed on our way toward a more sustainable and circular society.^2,3^ Besides the conceptually straightforward approach of phasing down the production of single use items, suitable conversion or recycling techniques can offer end-of-life options for plastic waste. However, only 14% by weight of plastic waste is recycled globally.^4^ One reason for low recycling rates is the lack of material quality obtained by the predominantly applied technique of melting and re-extrusion.^5−7^ Therefore, chemical recycling has been studied to produce monomers for remaking high-quality plastics, and other molecules to replace fossil resources.^7−10^ Pyrolysis as a chemical recycling method relies on heating the plastic in an inert atmosphere to induce cracking into smaller compounds. For the most produced polymers, polyethylene (PE) and polypropylene (PP), however, controlling the cracking selectivity is difficult due to random bond scission and further reactions, leading to a low-value mixture of hundreds of different hydrocarbons.^11^ Low-temperature strategies are a promising alternative. In contrast to pyrolysis, they could slow down unselective follow-up reactions of intermediates, thereby allowing better control over reactivity pathways.

While thermal cracking of PP and PE starts above 275 and 330 °C, respectively,^12^ Sohma et al. showed that polymer backbone bonds can be homolytically cleaved leading to organic radicals even at −196 °C when these polymers are exposed to mechanical force.^13^ While mechanochemical degradation is treated as an unwanted effect in polymer processing and recycling, it has recently been exploited for the purposeful conversion to monomers.^14^ Recent efforts toward mechanochemical degradation involve newly designed polymers and novel synthesis methods.^15,16^ Examples for the mechanochemical recycling of realistic bulk polymer streams are the depolymerization of polystyrene (PS), poly(α-methylstyrene), and poly(methyl methacrylate).^17−20^ These polymers have favorably high glass transition temperatures *T*_g_ to enable facile chain cleavage,^21^ and low ceiling temperatures *T*_c_ providing a thermodynamic driving force for depolymerization.^18^ In contrast, PE and PP are more challenging to depolymerize due to their low *T*_g_ and high *T*_c_.^14,22^ Thus, it is not surprising that their additive-free mechanochemical depolymerization is, to the best of our knowledge, not yet reported.

Ball milling is conventionally used for grinding, but also causes mechanochemical transformations in solids,^23−25^ and is widely explored for material synthesis.^26^ In the case of mechano-catalysis, a catalyst is usually added as a separate component, such as a powder.^27,28^ However, separating solid reactants/products from a catalyst powder is challenging,^29^ and exposing substrates to mechanical forces and a solid catalyst at the same time is not trivial. Therefore, direct mechano-catalysis has evolved to optimize contact between substrate and catalyst, relying on grinding tools which are catalytically active themselves,^30^ such as copper^31^ or palladium^32^ spheres. Current strategies are limited to metallic materials, utilizing their natural activity in, e.g., coupling and (de)hydrogenation reactions.^29^ In contrast, the purposeful acidic or redox functionalization of ceramic grinding spheres is, to the best of our knowledge, not yet reported, prohibiting an application of this concept to a broader reaction space.

Here, we explore the direct mechano-catalytic depolymerization of plastic waste without additional heating, below 50 °C, and at ambient pressure. Instead of adding a catalyst material as powder or using catalytically active metal grinding spheres, we purposefully surface-activated ceramic grinding spheres to create catalytic sites, which enhance depolymerization. We name these materials surface-activated mechano-catalysts (in short, SAM catalysts).

## Results and Discussion

## Conversion of Polypropylene at Ambient Temperature

**Figure 1:**
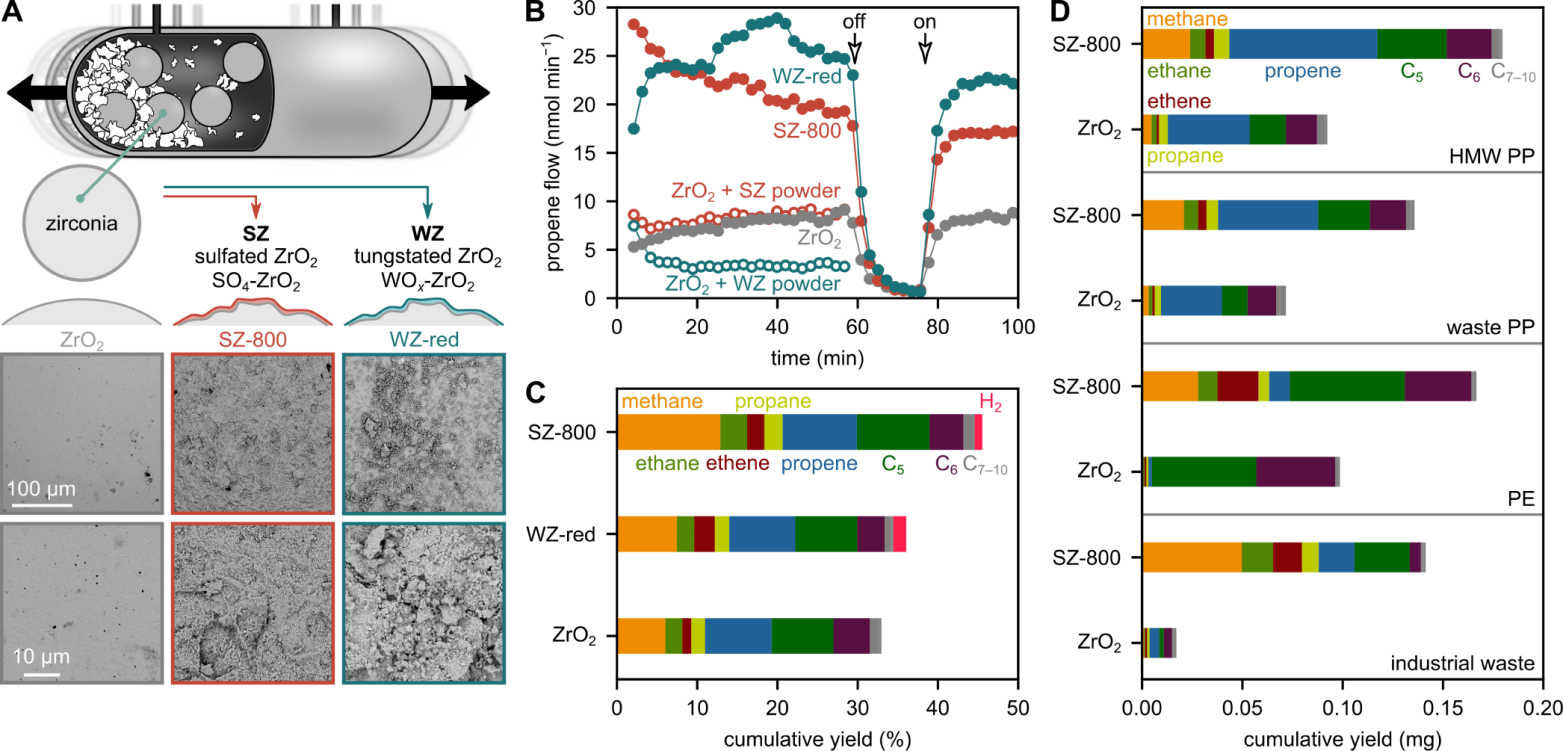
Mechano-catalytic conversion of plastic (waste) to C_1–10_ hydrocarbons. (A) Milling container, surface activation strategy, and SEM images of grinding spheres. (B) Propene flow during milling of 2 g model PP with sulfated (SZ-800), tungstated (WZ-red), and untreated (ZrO_2_) spheres at 30 Hz, compared to milling with untreated spheres and 0.1 g SZ and 0.2 g WZ powder catalysts. The shaking was turned off/on at the indicated points. (C) Cumulative yields during milling of 20 mg model PP for 1 h at 35 Hz with sulfated (SZ-800), tungstated (WZ-red), and untreated (ZrO_2_) spheres. (D) Cumulative yields during milling of HMW PP (2 g, 30 Hz), waste PP (2 g, 30 Hz), PE (2 g, 35 Hz) and industrial waste (1 g, 30 Hz) for 1 h with sulfated (SZ-800) and untreated (ZrO_2_) spheres.

We used a shaker mill (Figure 1A) to investigate the mechanochemical and direct mechano-catalytic conversion of plastic waste into small hydrocarbons. The container was typically filled with 2 g of plastic (typically PP), 5 grinding spheres (diameter = 10 mm), and shaken at 30 Hz at room temperature (see Figure S1 for different frequencies and filling degrees). Products were eluted from the container using a constant flow of N_2_ through a gas inlet and outlet, and analyzed using an online gas chromatograph.

Milling of model PP with untreated zirconia grinding spheres generates a steady stream of gaseous hydrocarbons, such as propene, C_5_, and C_6_ hydrocarbons (Figures 1, S1 and S2). During milling, the container temperature rises to 40 °C due to friction and mechanical energy dissipation (Figure S3). Hydrocarbon production immediately stops and starts again upon switching off and restarting the milling, while the temperature declines only slowly. The formation of hydrocarbons is, therefore, directly linked to local mechanochemical effects rather than an increase in bulk temperature. Furthermore, PP after milling is still a powder (Figure S4), indicating that the depolymerization is not induced by bulk heating. While pyrolysis at high temperatures leads to hundreds of different long-chained hydrocarbons with low selectivity,^11^ we detected typically less than 30 in the gas phase, the majority of them below C_7_ (see Figure S5 for chromatogram of higher nonvolatile products). In addition, several of the observed products, such as methane, ethane, and propane, are more saturated than the starting material PP, which indicates the presence of more dehydrogenated species in the milled residue. During milling, the crystallinity of PP slightly declines from 45 to 42% indicating amorphization of crystalline domains due to repeated mechanical impact (Figure S6).

To increase product yields and selectivities, we purposefully functionalized the grinding spheres with catalytic sites, which leads to surface-activated mechano-catalysts (in short, SAM catalysts). This was achieved by sulfuric acid treatment at 800 °C (SZ-800) or tungstation (WZ-red) of commercial zirconia spheres. In the first strategy, sulfuric acid etches the smooth surface of grinding spheres and causes chemical changes (Figures 1A and S7–S9). In the second activation strategy, we first etched the grinding spheres with molten NaOH after which we immobilized tungstate species from an aqueous solution. Finally, the spheres were calcined and reduced (Figures 1A and S10–S12).

Milling with SAM catalysts increases the hydrocarbon production. Surprisingly, milling with powder catalysts does not lead to an increase (Figures 1B and S13), although the total active surface areas of added SZ and WZ powders are 3–4 orders of magnitude larger than the external surface areas of five grinding spheres, without accounting for surface roughness (see methods section for calculation). We believe that this activity gap between SAM catalysts and conventional powders is caused by the direct localization of catalytic sites at the impact zone, i.e., where the mechanical energy input is the greatest. In contrast, the likelihood that a catalyst powder that is finely dispersed in the milling mixture is trapped at the impact site of the grinding spheres together with the polymer is low. Milling with optimized parameters, i.e., shaking frequency and filling degree, leads to 46 and 36% C_1–10_ hydrocarbons and H_2_ after 1 h with sulfated and tungstated grinding spheres, respectively (Figure 1C), while only 33% are obtained with untreated spheres. On the other hand, only <0.05% of hydrocarbons are obtained when using less ideal milling parameters, such as higher filling degrees and lower frequencies (see Figures S1 and S14 for other filling degrees and frequencies).

We furthermore tested the broader applicability of SAM catalytic plastic conversion. Sulfated zirconia also catalyzes the degradation of a high molecular weight (HMW) PP, waste PP, PE, PS, and even an industrial waste sample (Figures 1D and S15). Similar to PP, milling of PE and PS with SAM catalysts leads to an increased formation of their monomers ethene and styrene, respectively.

During milling of model PP with SAM catalysts, hydrocarbon productivity declines due to the abrasion of active surface sites over the course of ca. 5 h caused by vigorous collisions between spheres and the hard walls of the grinding vessel (Figure S16). Abrasion and flattening of surfaces can be observed via SEM (Figure S17) and the naked eye. Further research is, therefore, needed regarding the abrasion resistance of surface-activated spheres and identifying materials that are mechano-catalysts in their bulk form to maintain activity even with abrasion. However, the reactivation of sulfated grinding spheres is facile; resubjecting them to the synthesis treatment restores original activity without any declining trend over three runs (Figure S18).

## Radicals Are Intermediates for the Mechanochemical Formation of Small Hydrocarbons

**Scheme 1:**
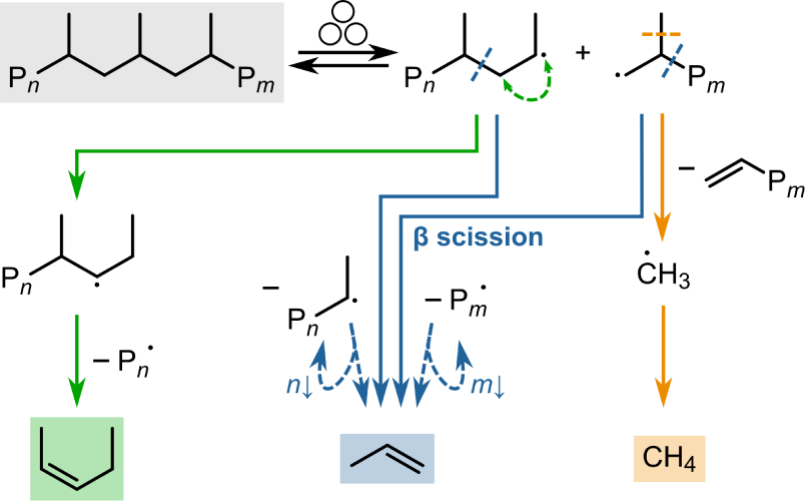
Simplified Reaction Scheme of the Radical Reactions toward Observed Hydrocarbons from PP Chain scission via ball milling leads to mechano-radicals. These can undergo further scission to smaller molecules, such as propene via β scission, methane, and higher products after hydrogen transfer.

**Figure 2:**
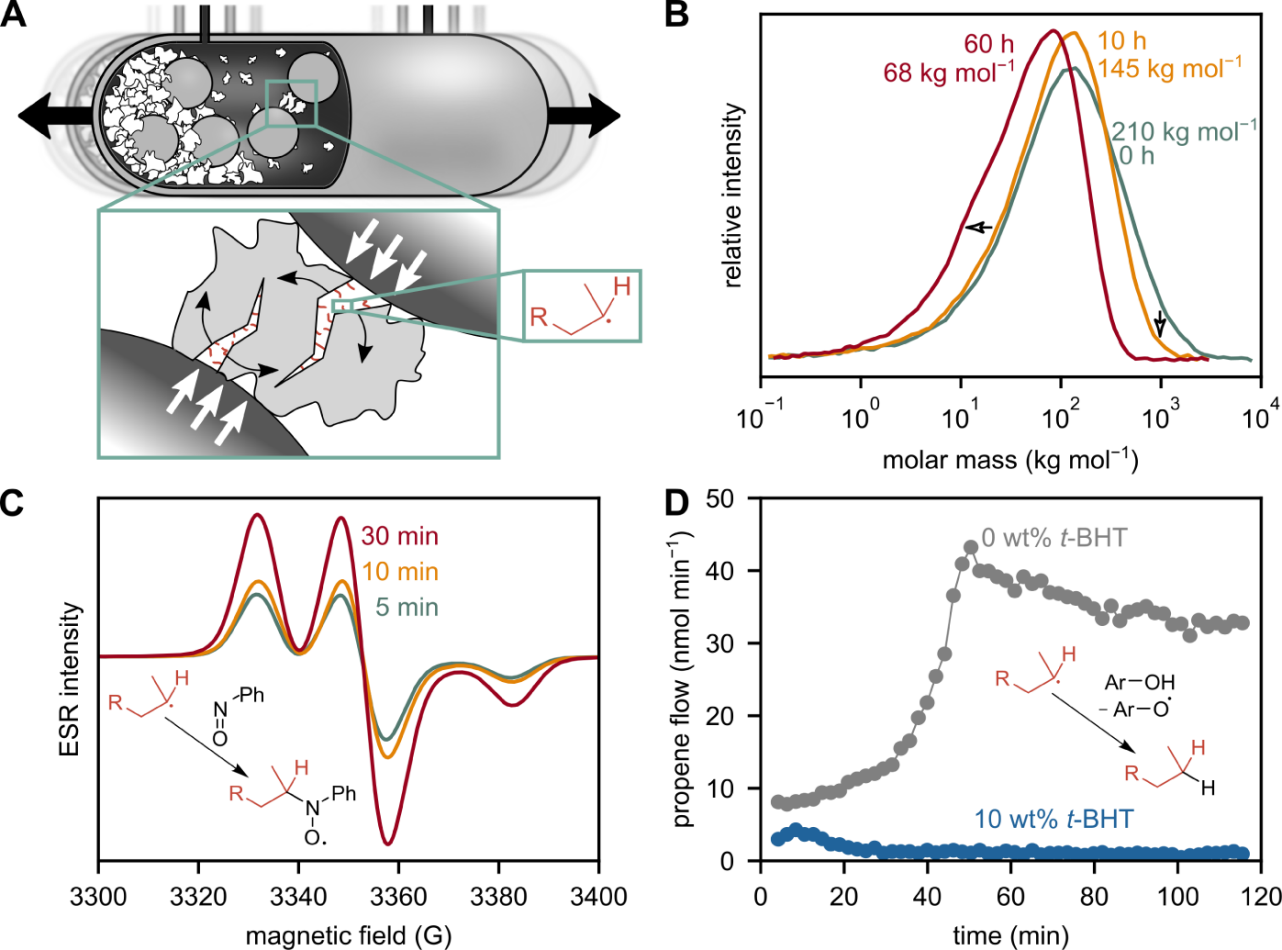
Mechanochemical conversion of PP with untreated grinding spheres proceeds via radicals. (A) Milling chamber, fracture mechanism, and mechano-radical formation. (B) Molar mass distributions before and after milling waste PP for 10 and 60 h with untreated ZrO_2_ spheres. (C) Mass-normalized ESR spectra after milling model PP with 50 mg nitrosobenzene for 5, 10, and 30 min. (D) Quenching of propene formation with 10 wt % *t*-BHT, while milling HMW PP with untreated ZrO_2_ spheres.

The mechanochemical conversion of PP with untreated grinding spheres yields C_1–10_ hydrocarbons (Figure 2A). We propose a mechanistic model based on radical chemistry (Scheme 1).^14^ The mechanochemical activation step is the homolytic backbone cleavage of polymer chains leading to mechano-radicals. These can undergo β scission causing direct depolymerization to the monomer propene, restoring the radical functionality on the active chain end. Radical transfer reactions and subsequent scissions yield more complex products, such as C_5_. Methane could be formed via highly reactive methyl radicals and subsequent hydrogen abstraction. Due to their low stability, the formation of methyl radicals is much more energetically challenging than, e.g., β scission to propene.^33^ The fact that methane is still observed illustrates the high local energy densities present under mechanochemical activation. Termination of active radicals can occur via disproportionation or radical combination. Both could lead to branching and cross-linking of the chains. The latter was probed via thermogravimetric analysis (TGA), but not observed (Figure S19).

We followed the evolution of polymer chain length via high-temperature gel permeation chromatography (GPC, Figure 2B), observed radicals using electron spin resonance (ESR, Figure 2C), and quenched product formation with a radical scavenger (Figure 2D).

Milling waste PP for 10 and 60 h lowers the weight-average molar mass (*M*_w_) from initially 210,000 to 145,000 and 68,000 g mol^–1^ (Table S1). Interestingly, cleavage proceeds more readily for higher molecular weight chains (Figure 2B, see Figure S20 and Table S2 for comparison of different PP types). This behavior can be attributed to a more effective force transferal for longer chains and is in line with typical polymer mechanochemical models, which also hypothesize preferential breakage in the middle of the chain.^34,35^ This is an important distinction from thermo-chemical activation via cracking or “hot spots”, where more random cleavage along all chains would be expected. The observed cleavage locations therefore point toward a mechanochemical activation motif in the case of ball milling PP.^36−38^

We investigated mechano-radicals via ESR spectroscopy, which is very sensitive for systems with unpaired electrons, e.g., organic radicals. Due to their high reactivity and transient nature, carbon-centered radicals could not be tracked directly, but their formation was indirectly evidenced by using nitrosobenzene as a spin trap to form stable nitroxide adducts (Figure 2C). Relative radical concentrations obtained by 2-fold integration of the shown derivative spectra indicate a continuous formation of mechano-radicals by chain scission (Figure S21).

To test the role of mechano-radicals as key intermediates, we milled HMW PP together with a radical scavenger (pentaerythritol tetrakis(3,5-di-*tert*-butyl-4-hydrocinnamate), abbreviated as *t*-BHT). Capturing radicals quenches product formation almost entirely (Figures 2D and S22). The quenching of all product formation with a radical scavenger implies that radicals with a sufficient lifetime are integral intermediates, and that there are no additional pathways to products that do not involve radical reactions.

## Mechano-Catalysis beyond Acidic Cracking

**Figure 3:**
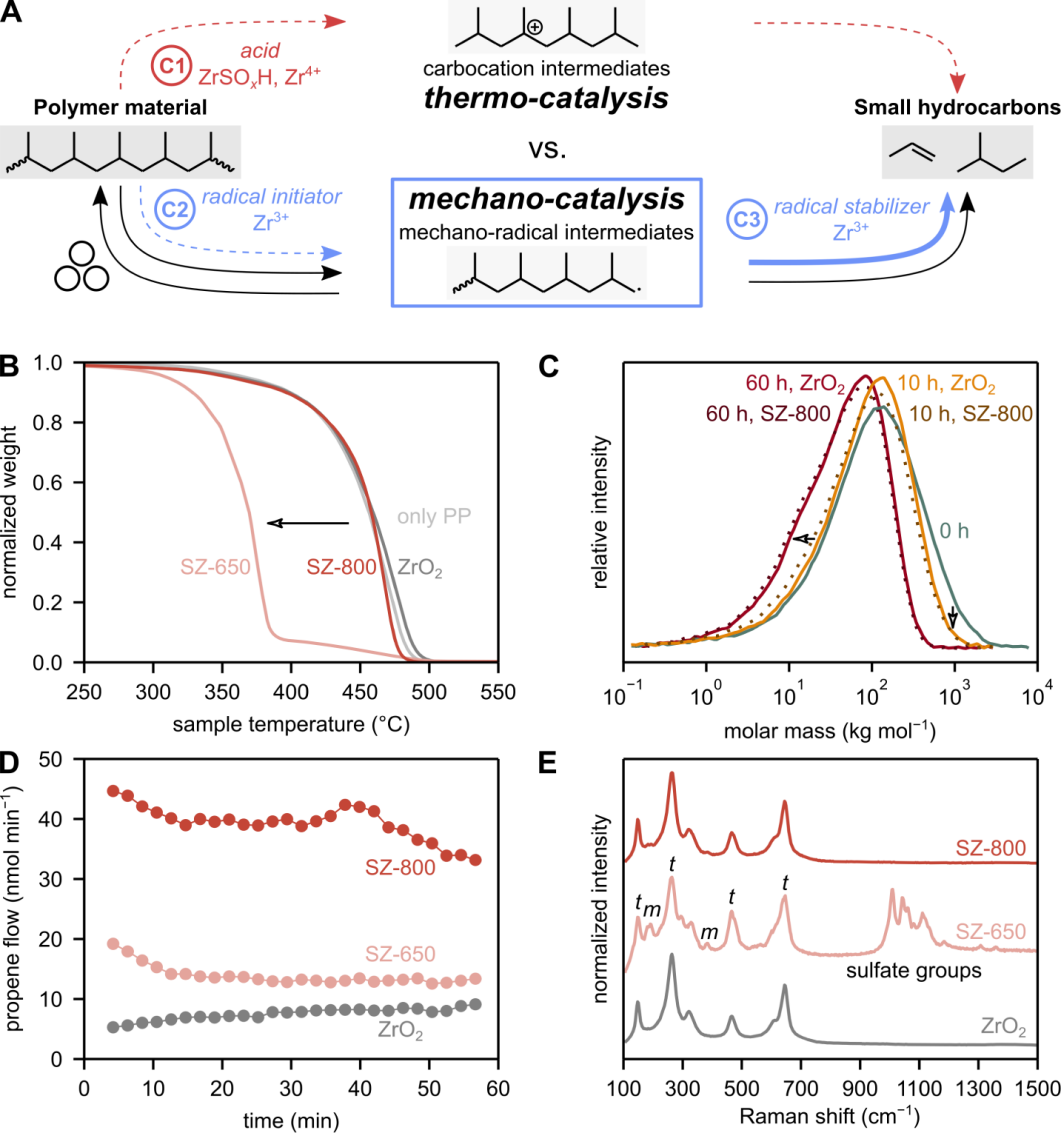
Mismatch between thermo- and mechano-catalysis. (A) Hypothetical catalytic pathways (C1–C3) to explain how a catalyst causes an increase in the formation of small hydrocarbons during ball milling. Noncatalytic, purely mechanochemical pathways are indicated with black arrows. (B) TGA profiles of model PP with sulfated (SZ), and untreated (ZrO_2_) grinding spheres. (C) Molar mass distributions before and after milling waste PP with sulfated (SZ-800), and untreated (ZrO_2_) grinding spheres for 10 and 60 h. (D) Propene formation during ball milling of model PP with sulfated (SZ), and untreated (ZrO_2_) grinding spheres. (E) Raman spectra of sulfated (SZ), and untreated (ZrO_2_) grinding spheres. Signals corresponding to monoclinic (m) and tetragonal (t) ZrO_2_ are indicated. The surface of SZ-650 spheres is heterogeneous, and obtained spectra vary from spot to spot (Figure S24).

Milling with SAM catalysts boosts the formation of small hydrocarbons during milling. Our initial aim was to provide acid sites for cracking and depolymerization, drawing inspiration from classical thermo-catalytic hydrocarbon conversion mechanisms (C1 in Figure 3A).^39^ However, considering that radicals are the key intermediates without a catalyst (Figure 2), an alternative hypothesis is that the catalyst promotes the radical pathway (C2 and C3).

Typical thermo-catalytic cracking of polyolefins is governed by cations, and reactivity is mediated by the surface acidity of the catalyst. We used TGA (Figure 3B, see Figure S23 for reference profiles) of model PP under N_2_ atmosphere to investigate the thermo-catalytic activity of SAM catalysts. In TGA, weight loss indicates the cracking of the plastic, with an earlier weight loss indicating higher catalytic cracking activity. SZ spheres are catalytically active as they crack PP at a lower temperature than untreated zirconia. In addition, it has been speculated that acidic zirconia sites can catalyze organic transformations during ball milling.^40^ Therefore, it is plausible that acidic sulfated zirconia sites catalyze thermal cracking of PP in the ball mill. While this could unlock synergies between thermal and mechano-catalytic effects, we believe that this is not the cause for the increase in product formation during ball milling based on three observations: (i) the temperature reached during ball milling is far lower than the cracking temperature. (ii) The active sites on SAM catalysts do not promote backbone cleavage. (iii) There is a reactivity mismatch between thermo- and mechano-catalytic conversion.

Regarding (i): the temperature reached during ball milling is far lower than the cracking temperature (onset at 250 °C, Figure 3B). Bulk temperatures in the ball mill reach only ca. 40 °C due to friction and energy dissipation (Figure S3). Temperature heterogeneities and transient high-energy environments (“hot spots”) could cause higher local temperatures. However, molecular dynamics simulations on polyethylene have shown that shock loads of >1000 m s^–1^ would be necessary for local temperatures to approach cracking temperatures,^41^ while grinding sphere velocities in similar configurations as ours are <10 m s^–1^.^37,42^ It is therefore unlikely that the PP melting point of 160 °C, let alone the cracking temperature of 250 °C, were systematically exceeded during milling.

Regarding (ii): the active sites on SAM catalysts do not promote backbone cleavage. The molar mass distributions after milling waste PP for 10 or 60 h with catalytic and untreated spheres show no significant differences (Figure 3C). If the catalyst were to thermo-catalytically crack backbones, this would be reflected in a lower molar mass after milling with catalytic grinding spheres compared to untreated spheres.

Regarding (iii): there is a reactivity mismatch between thermo- and mechano-catalytic conversion. In the case of sulfated zirconia, mechano- and thermo-catalytic activities are highly dependent on the exact synthesis procedure. SZ-650 and SZ-800 were prepared by sulfuric acid treatment at 650 and 800 °C, respectively. The catalyst prepared at a lower temperature (SZ-650) is a highly thermo-catalytically active material, while SZ-800 is not (Figure 3B). Thermo-catalytic activity of sulfated zirconia is typically governed by acid sites connected to sulfate species. Indeed, SZ-650 displays a much higher loading of sulfur (EDX in Figure S9) and sulfate groups compared to SZ-800, as visible in Raman spectroscopy (1000–1200 cm^–1^, Figure 3E) and TGA–mass spectrometry measurements (Figure S25). However, the higher sulfate loading, acid site density, and thermo-catalytic activity for SZ-650 do not lead to a higher mechano-catalytic activity. Instead, propene formation during ball milling is higher for SZ-800 than for SZ-650 (Figure 3D). This mismatch highlights fundamental differences in the activation mechanism between thermo- and mechano-catalysis. The increase in small hydrocarbon formation during ball milling is caused by a mechanism beyond classical cracking over acid sites.

## Mechano-Catalysis Controls the Reactivity of Mechano-Radicals

Our SAM catalysts seem to lead to a higher product formation via a pathway fundamentally different from acid-based thermo-catalysis. The mechanism appears to proceed via radicals instead of cations. We found that product formation is completely quenched when milling HMW PP with SZ-800 in the presence of *t*-BHT (Figure S22). Therefore, all generated products, even in the catalytic case, originate from radicals as key intermediates, and there is no pathway that does not, at least at some stage, involve radical chemistry.

The catalyst could lead to higher product formation via the following pathways: (i) causing additional homolytic chain cleavage events, or act as a radical starter in another way (pathway C2 in Figure 3A). This would lead to more backbone cleavage when milling with catalytic spheres compared to untreated spheres, and would lead to a more pronounced decrease in molar mass. However, this is not evident from our experiments (Figure 3C). (ii) We therefore propose that the SAM catalyst stabilizes/interacts with mechano-radicals previously generated purely by mechanical forces (pathway C3 in Figure 3A). This could operate along the lines of controlled radical (de)polymerization by forming reversible adducts with active chain ends.^43^ This adduct formation could decrease the concentration of free radicals in the bulk material, and thereby suppress their termination, leading to a longer lifetime. In addition, this stabilization could favor certain pathways of radical decomposition toward smaller hydrocarbons, thereby changing selectivity patterns.

**Figure 4:**
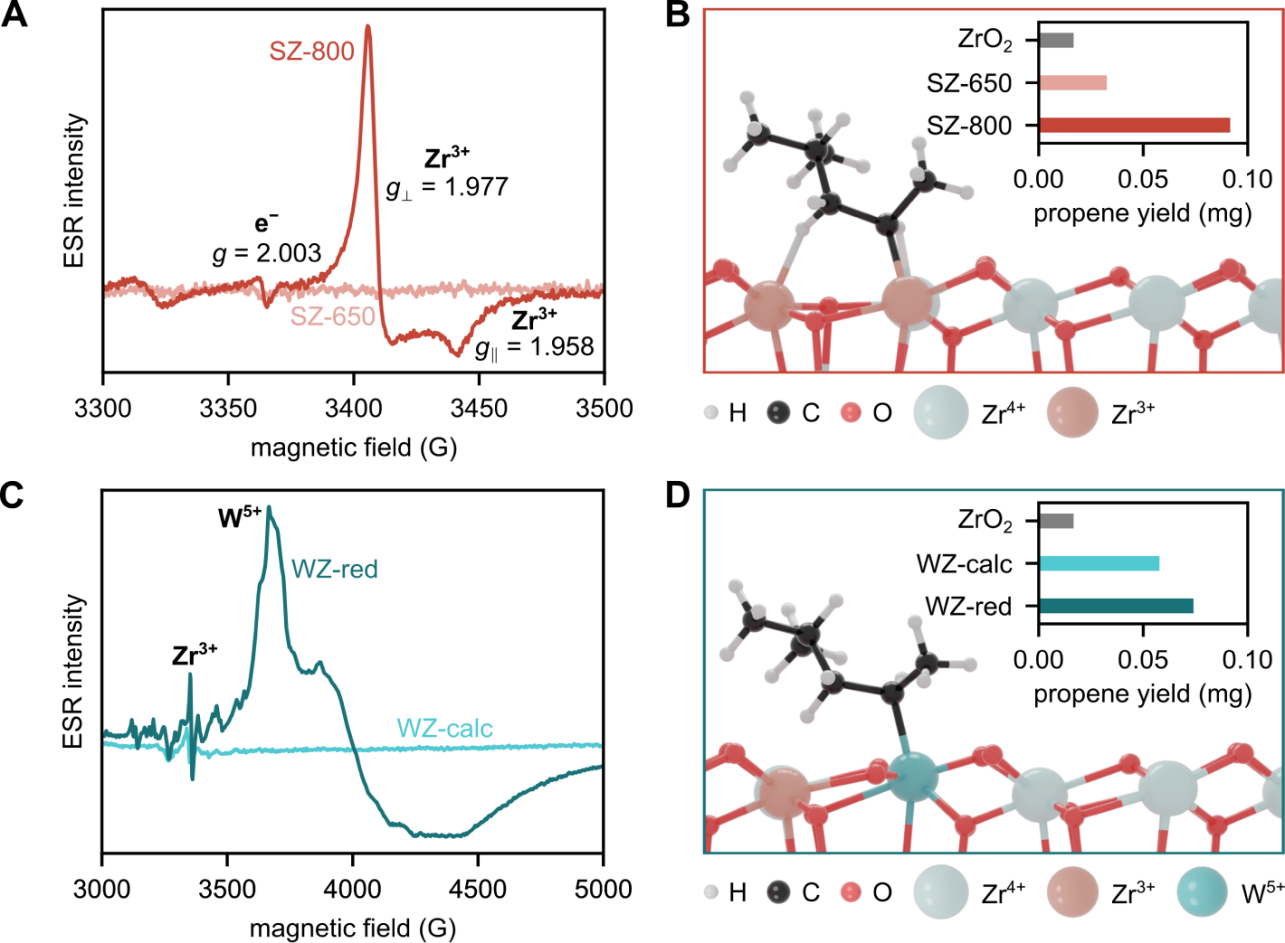
Interaction of catalytic spheres with mechano-radicals. (A) ESR spectra of SZ-650 and SZ-800. (B) Proposed adsorption geometry and stabilization of a secondary carbon-centered radical on SZ-800, and propene yields during 1 h milling of model PP at 30 Hz. (C) ESR spectra of WZ-calc and WZ-red. (D) Proposed adsorption geometry and stabilization of a secondary carbon-centered radical on WZ-red, and propene yields during 1 h milling of model PP at 30 Hz.

The interaction of the SAM catalyst with radicals could be rooted in the presence of unpaired electrons, and we used ESR spectroscopy to investigate the electronic structure of treated zirconia. Since grinding spheres did not fit into the ESR tubes, we used residual powders collected during the synthesis procedures as model systems. For tungstated zirconia, the powder that is immobilized on the spheres was used. For sulfated zirconia, we used sulfated zirconia powder etched off the spheres during the high-temperature treatment with sulfuric acid. In the case of SZ-800, a paramagnetic axial Zr^3+^ signal (*g*_⊥_ = 1.977 and *g*_∥_ = 1.958) and *F*-centers (*g* = 2.003), i.e., electrons in oxygen vacancies, were observed (Figure 4A, Table S3).^44^ The formation of such species due to a high-temperature vacuum treatment of zirconia or due to heating of sulfated zirconia has been reported,^45−47^ leading to a net reduction of Zr^4+^ to Zr^3+^. The zirconia grinding spheres we used were stabilized with Y_2_O_3_, which increases the amount of oxygen vacancies and could assist the formation of Zr^3+^ species and *F*-centers under suitable conditions. Hypothetically, both Zr^3+^ species and *F*-centers could interact with and stabilize mechano-radicals, which would result in their increased lifetime, or direct the selectivity from radicals toward small hydrocarbons. To this end, these catalyst species must be able to display radical-like reactivity, which indeed seems to be the case; highly reactive Zr^3+^ radical cations in polymorphous zirconia can homolytically dissociate H_2_,^48^ and the reactivity of sulfated zirconia with organic substrates is connected to accepting single electrons as part of a radical cation mechanism.^49,50^ It is therefore plausible for Zr^3+^ centers to interact with and stabilize mechano-radicals H*_y_*C*_x_*^•^ via an adsorption equilibrium H*_y_*C*_x_*^•^ + Zr^III^ ⇄ H_*y*_C_*x*_–Zr^IV^, in which Zr changes its oxidation state and hence acts as a redox-active material. While SZ-800 features a prominent Zr^3+^ signal, such a signal is absent for SZ-650, and we hypothesize that the higher concentration of Zr^3+^ critically enhances the ability of SZ-800 to promote the radical pathway, rendering it a better mechano-catalyst than SZ-650 (Figure 4B). For tungstated zirconia, we investigated the material before and after reduction in H_2_ (Figure 4C). Both featured Zr^3+^, but its concentration was enhanced during the reduction step, which corresponds to a higher mechano-catalytic activity after reduction (Figure 4D). In addition, we observed W^5+^ species in the reduced sample.^51,52^

To rationalize the interaction of carbon-centered mechano-radical intermediates with sulfated and tungstated tetragonal and monoclinic zirconia surfaces, we performed density functional theory (DFT) calculations.^53−59^ We modeled sufficiently relaxed surfaces of SZ-650 and SZ-800 based on experimental evidence, such as Raman and ESR spectroscopy, and TGA-MS measurements. For SZ-650, zirconia surfaces containing sulfate groups as the dominant species were investigated, while we created oxygen vacancies (V_O_) to produce Zr^3+^ ions for modeling SZ-800. The adsorption energies of carbon-centered radicals on these surfaces are indicators of possible interactions, and highly dependent on the exact nature of surface species (Table 1 for tetragonal ZrO_2_, Table S4 for monoclinic ZrO_2_, Tables S5–S8 for images). While the incorporation of SO_4_ or Brønsted acid sites leads to virtually no stabilization of radical intermediates, oxygen vacancies and Zr^3+^ cations stabilize mechano-radicals by ca. 2 eV, regardless of the presence of SO_4_. This enhanced interaction explains why the Zr^3+^-rich SZ-800 spheres promote the radical pathway more effectively than the Zr^3+^-poor SZ-650 spheres and show a higher catalytic activity, especially to propene (Figure 3D). Adsorption energies also help rationalize the promotion over tungstated zirconia, which was modeled as Zr^3+^–O–W^5+^ species.

**Table 1:** Adsorption Energies of Carbon-Centered Primary and Secondary Radicals by DFT Calculations on Tetragonal ZrO_2_ Surfaces

	*E*_ads_/eV	
surface species	primary radical	secondary radical
pristine	–0.74	–0.65
SO_4_	–0.69	–0.56
Zr^3+^–V_O_	–2.79	–2.72
SO_4_ & Zr^3+^–V_O_	–2.82	–2.49
Zr^3+^–O–W^5+^	–2.28	–1.99

The stabilization of radical intermediates is helpful in guiding their reactivity and hampering recombination. However, too high adsorption energies would excessively decrease reactivity, ultimately leading to low depolymerization rates. In that sense, the value of 2 eV represents quite strong interactions between radicals and surface species. However, this value was calculated under static conditions, not accounting for the vigorous mechanical agitation in the ball mill, and two important factors which shift the adsorption equilibrium to the dissociated state have to be accounted for: (i) adsorbed species could be tribo-chemically activated by impact forces. (ii) The Zr–polymer adduct can be mechanochemically cleaved, just like polymer backbones. Both mechanisms could activate the adsorbed species under mechanical agitation, despite stronger adsorption in the calculated resting state.

## Conclusions

While thermal cracking of polypropylene only starts at reaction temperatures above 250 °C, we demonstrate that it can already be depolymerized to its monomer propene and other products at ambient temperature. This is the result of mechanochemical homolytic C–C bond cleavage and follow-up reactions of formed radicals. We introduce the concept of functionalizing ceramic grinding spheres, making them surface-activated mechano-catalysts (SAM catalysts). During milling of polypropylene, their redox-active surface sites likely interact with mechano-radicals and favor the production of small hydrocarbons over termination reactions. Furthermore, the functionalized grinding spheres could be used to catalyze other processes that require redox functionality, beyond depolymerization, in the future.

Mechanochemical bond scission of polymers is usually undesired in polymer processing, such as during extrusion, but we show that it can be exploited for chemical recycling at ambient temperature. Chemical conversion in the ball mill has the potential advantage of tapping renewable sources of mechanical energy, such as hydropower and wind energy, directly without prior inefficient conversion into electrical and/or thermal energy. Our mechano-catalytic approach offers a new dimension for polyolefin recycling beyond conventional cracking catalysis that requires less energy and is more selective. However, space–time yields, especially when loading high amounts of plastic material, have to be drastically increased before reaching industrial viability. Industrial scale ball mills exist in the cement industry and could be used to upscale the technology, and we expect an increase in yield when using larger and heavier grinding spheres.

## Data Availability

Data and code used in the analysis are available via the Yoda repository (10.24416/UU01-TLREG2).

## Supporting Information Available

Experimental details regarding ball milling experiments, catalyst synthesis, and characterization, besides additional figures for hydrocarbon production, temperature profiles, electron micrographs, ESR and Raman spectra, TGA profiles, gas chromatograms, photographs, besides additional tables for GPC data and DFT calculations (PDF)
