# Supplementary material for: Surface-Activated Mechano-Catalysis for Ambient Conversion of Plastic Waste
Source: J Am Chem Soc. 2024 Sep 9;146(38):26139–47. doi: 10.1021/jacs.4c07157 (PMC11440499; doi:10.1021/jacs.4c07157)
Supplement: Supplementary file 1 — ja4c07157_si_001.pdf [file ja4c07157_si_001.pdf]

Supporting Information for

Surface-activated mechano-catalysis for ambient conversion of plastic waste

Adrian H. Hergesell[1], Renate J. Baarslag[1]‡, Claire L. Seitzinger[1]‡, Raghavendra Meena[2,3], Patrick Schara[4], Željko Tomović[4], Guanna Li[2], Bert M. Weckhuysen[1], Ina Vollmer[1]*

[1]Inorganic Chemistry and Catalysis Group, Institute for Sustainable and Circular Chemistry, Utrecht University; 3584 CG Utrecht, The Netherlands.

[2]Biobased Chemistry and Technology, Wageningen University; 6708 WG Wageningen, The Netherlands.

[3]Laboratory of Organic Chemistry, Wageningen University; 6708 WE Wageningen, The Netherlands.

[4]Polymer Performance Materials Group, Department of Chemical Engineering and Chemistry, Eindhoven University of Technology; 5600 MB Eindhoven, The Netherlands.

*Corresponding author. Email: i.vollmer@uu.nl

‡These authors contributed equally to this work.

Experimental details

Ball milling experiments

We investigated the mechano-catalytic conversion of six plastic materials (see **Table S1** for information) into small hydrocarbons. Polypropylene (PP) materials and polystyrene (PS) were either commercially available polymer products (Sigma-Aldrich, Ducor Petrochemicals) or sourced from a food container. The polyethylene (PE, ultra-high molecular weight, UHMW) sample was kindly donated by Avient Protective Materials (Geleen, the Netherlands). The industrial waste fraction was sourced from industrial packaging waste, containing mainly PE/PP foils with ca. 20% polyamide, and fractions of polyethylene terephthalate, ethylene vinyl alcohol and PS.

In a typical ball milling experiment, 2 g plastic (+ potential additives) were loaded into a 25 ml tungsten carbide ball milling container (Retsch, 94% WC and 6% Co) and five ZrO_2 grinding spheres (94.5% ZrO_2 and 5.2% Y_2O_3) of 10 mm diameter were added. The container with a Teflon seal was closed hand-tight and shaken using a Retsch MM 500 vario mixer mill for a given time at a frequency of typically 30 Hz, after which the milled material was collected for further analyses. In between experiments, the container with gas ports was cleaned with water, acetone, and dried at 110 °C for > 2 h.

As additives, 50 mg nitrosobenzene (Sigma-Aldrich, 97%) for spin trapping were used together with 2 g model PP, while 200 mg *t*-BHT (pentaerythritol tetrakis-(3,5-di-*tert*-butyl-4-hydrocinnamate), Sigma-Aldrich, 98%) were used for radical quenching experiments together with 2 g HMW PP. As catalyst powders, 100 mg SZ powder and 200 mg WZ-red powder, both synthesized as described below, were used together with 2 g model PP. The total surface areas of added powder are 0.1 g × 100 m^2 g^{-1} = 10 m^2 for SZ powder and 0.2 g × 36 m^2 g^{-1} = 7.2 m^2 for WZ-red powder, while five spheres (10 mm diameter) have an external surface area of 0.0016 m^2, without accounting for surface roughness. This is a difference of 3–4 orders of magnitude.

For continuous gas analysis, holes were drilled into the commercial container via electrical discharge machining, and 1/8″ Swagelok connections were welded to it. Products were eluted from the milling chamber using a flow of 12.5 ml min^{-1} N_2 as a carrier gas and internal standard. Product analysis was performed using an online Global Analyser Solutions gas chromatograph (GC). For the detection of H_2 and N_2, this device was equipped with a thermal conductivity detector (TCD) coupled to a 2 m × 0.32 mm Rtx-1, 3.0u and a 3 m × 0.32 mm Carboxen1010 column. A flame ionization detector (FID) was used for the detection of hydrocarbons ($C_{1–3}$: 3 m × 0.32 mm Rtx-1, 3u column and 15 m × 0.32 mm Al_2O_3/Na_2SO_4 column; $C_{4–7}$: 2 m × 0.28 mm MXT-1, 1u column and 14 m × 0.28 mm MXT-1, 1u column; $C_{5–10}$: 2 m × 0.28 mm MXT-1, 0.5u column and 15 m × 0.28 mm MXT-1, 0.5u column). The flow of N_2 ($F_{\mathrm{N_2}}$ = 12.5 ml min^{-1} = const.) was used as an internal standard to counter the change in total volumetric flow ($F_{\mathrm{total},i} = \frac{F_{\mathrm{N_2}}}{y_{\mathrm{N_2},i}}$) caused by product formation. The molar concentration of N_2 at each injection during the run ($y_{\mathrm{N_2},i}$) was determined according to **Eq. 1** by using the average of the peak areas ($A_{\mathrm{N_2},i}$) of the first three injections prior to start of the reaction after the flow had stabilized.

$$y_{\mathrm{N_2},i} = \frac{A_{\mathrm{N_2},i}}{\frac{\sum_{i=-2}^{i=0} A_{\mathrm{N_2},i}}{3}} \cdot \; y_{N_{2,0}} \qquad (1)$$

The molar carbon flow caused by each hydrocarbon (C_xH_y) was determined according to **Eq. 2** by using the carbon number *x* of the respective hydrocarbon.

$$F_{\mathrm{C}_x\mathrm{H}_y,i} = y_{\mathrm{C}_x\mathrm{H}_y,i} \cdot F_{\mathrm{total}} \cdot x \qquad (2)$$

The concentration of each hydrocarbon ($y_{\mathrm{C}_x\mathrm{H}_y,i} = \frac{A_{\mathrm{C}_x\mathrm{H}_y,i}}{CF_{\mathrm{C}_x\mathrm{H}_y,i}}$) was determined by the ratio of peak area of this hydrocarbon ($A_{\mathrm{C}_x\mathrm{H}_y,i}$) and a calibration factor ($CF_{\mathrm{C}_x\mathrm{H}_y,i}$) determined by injection of a gas mixture with known content. $CF_{\mathrm{C}_x\mathrm{H}_y,i} = CF_{\mathrm{C}} \cdot x$, with CF_{C} as the calibration factor normalized by carbon number, which was determined by calibration with a mixture of methane, ethane, propane, butane, heptane and hexane. An FID response is approximately proportional to the carbon number.

The cumulative yield of a hydrocarbon could be calculated according to **Eq. 3** through integration of the molar flow over time (with $M_{\mathrm{C}_x\mathrm{H}_y}$ being the molecular weight of the hydrocarbon). Cumulative percentage yields are based on the initial amount of plastic.

$$Y_{\mathrm{C}_x\mathrm{H}_y}[\mathrm{g}] = \frac{M_{\mathrm{C}_x\mathrm{H}_y}}{x} \cdot \int_0^{t_{\mathrm{final}}} F_{\mathrm{C}_x\mathrm{H}_y,i}\, \mathrm{d}t \qquad (3)$$

Chromatograms on the C_{1-3} channel typically featured only methane, ethane, ethene, propane, and propene as C_{1-3} hydrocarbons. In the case of experiments with low filling degrees (20 mg PP), however, small contributions of additional compounds were observed (**Figure S26**). Their peaks were integrated and added to the ethene and propane integrals for further analysis.

Catalyst synthesis

Synthesis of functionalized zirconia grinding spheres was typically performed on either 3 mm (Retsch) or 10 mm (Laarmann) commercial zirconia grinding spheres. 10 mm Retsch spheres were used for the direct comparison of SZ-650 and SZ-800. For the **production of sulfated zirconia grinding spheres**, commercial zirconia grinding spheres were treated with concentrated sulfuric acid (H_2SO_4, VWR, 95%). To this end, self-made cylindrical quartz cups (internal diameter of 12 mm, height of 17 mm) and a self-made quartz cup holder were used (**Figure S27**). 10 mm grinding spheres were individually placed into the cups and submerged in 0.8 ml H_2SO_4 each. For the synthesis of 3 mm grinding spheres, eight spheres were placed into the cups, and submerged in 1.3 ml H_2SO_4. The holder with cups was placed inside a quartz tube (internal diameter of 28 mm, length of 67 cm) in a tubular oven (Thermolyne 79300 Tube Furnace, flow of 100 ml min^{-1} synthetic air) and heated to either 800 °C for 2 h (heating rate: 5 °C min^{-1}) to produce SZ-800 or to 650 °C for 5 h (heating rate: 2.5 °C min^{-1}) to produce SZ-650 spheres. After cooling to room temperature, the grinding spheres were separated from the residual powder in the cup. The spheres washed with deionized water until pH neutrality and dried in air, while the powder was collected for further analyses.

For the **synthesis of a sulfated zirconia powder catalyst (SZ powder)**, $Zr(OH)_4$ was impregnated with H_2SO_4 and then calcined. For the synthesis of $Zr(OH)_4$, a solution of 5.00 g $ZrOCl_2 \cdot 8H_2O$ in 50.00 g H_2O was prepared, and NH_4OH (Merck, 25% solution in water) was added until pH 9 was reached. The resulting suspension was filtered and the white precipitate was washed with H_2O until no chloride ions were detected in the filtrate by using $AgNO_3$. To test this after each washing step, 1 ml filtrate and 1 ml of a solution of 3.90 g HNO_3 (VWR, 65%,) in 17.23 g H_2O were mixed, and a few drops of a solution of 0.20 g $AgNO_3$ (Fisher Scientific, laboratory reagent grade) in 23.55 g H_2O were added. After no precipitate was observed anymore upon addition of the $AgNO_3$ solution, the white filter residue ($Zr(OH)_4$) was dried at 110 °C for 21 h in air. To generate sulfated zirconia, the obtained $Zr(OH)_4$ was added to 30 ml of a solution of 1.56 g H_2SO_4 (VWR, 95%) in 29.16 g H_2O and stirred for 20 min. The suspension was filtered

under vacuum, after which the residue was washed with H_2O, dried at 110 °C for 22 h in air, and calcined at 600 °C for 3 h (heating rate: 2.5 °C min^{-1}) in static air to obtain 1.29 g of sulfated zirconia powder. (Brunauer–Emmett–Teller surface area of 100 m^2 g^{-1}).

For the production of **tungstated zirconia grinding spheres**, commercial spheres were first etched with NaOH (Merck, 99%, enough for all to be fully submerged in molten NaOH) in a crucible to 425 °C for 3 h (heating rate: 2 °C min^{-1}) in static air. After cooling to room temperature, the NaOH was removed by solubilizing it in deionized water, and the grinding spheres were washed with water until pH neutrality and dried in air. Afterwards, a suspension of 2 g $Zr(OH)_4$ (Sigma-Aldrich, 97%) in 30 g H_2O was stirred for 1 h at 50 °C. To prepare the tungsten precursor, under stirring, 20 drops of NH_4OH (Merck, 25% solution in water) were added to a solution of 0.37 g $(NH_4)_6H_2W_{12}O_{40} \cdot xH_2O$ (Sigma-Aldrich, 72.8% tungsten, 99.99% trace metal basis) in 20 g H_2O until pH 10 was reached, after which the solution was stirred for 0.5 h. The tungsten-containing solution was added to the zirconium-containing suspension, and 10 grinding spheres were added. After stirring for 1 h at 60 °C, the grinding spheres were separated from the suspension, and both were used further: The spheres were dried in air for 1 h at 120 °C, and calcined (heating program: 1 °C min^{-1} to 200 °C, 1 h at 200 °C, then 2 °C min^{-1} to 700 °C, 3 h at 700 °C) in static air to produce WZ-calc spheres. The remaining suspension after removal of the spheres was filtered, and the residue was dried for 1 h at 110 °C. The resulting powder was calcined (heating program: 1 °C min^{-1} to 200 °C, 1 h at 200 °C, then 2 °C min^{-1} to 700 °C, 3 h at 700 °C) in static air to obtain **tungstated zirconia powder**. Subsequent reductions were performed in an oven using a quartz tube and a flow of 25% H_2 in N_2 for 1 h at 450 °C (heating rate: 5 °C min^{-1}) to produce WZ-red spheres or powder. (Brunauer–Emmett–Teller surface area of 36 m^2 g^{-1}).

Characterization

Electron spin resonance (ESR) measurements were performed in continuous wave on an X-band Bruker EMXplus instrument. For spin trapping experiments on model PP, 50 mg nitrosobenzene (Sigma-Aldrich, 97%) was added to the milling container in addition to the PP. After milling, ca. 70–80 mg of milled material was filled into a quartz tube and measured at room temperature at a microwave frequency of ca. 9.4 GHz and a modulation frequency of 100 kHz. The reported spectra are normalized by mass. The relative concentrations of trapped radicals are reported in arbitrary units and obtained by double cumulative summation (integration) of the mass-normalized ESR spectra in the magnetic field region between 3300 and 3400 G. Mass-normalized spectra of catalyst powders were obtained analogously, but at 99 K instead of room temperature. Since 3 and 10 mm spheres did not fit into the ESR tubes, we used powder residues collected after synthesis as model systems. For tungstated zirconia, we used the powder that is usually immobilized on zirconia spheres before and after reduction, and for sulfated zirconia, we characterized the residue obtained during the sulfation procedure.

Thermogravimetric analysis (TGA) was performed on a Perkin Elmer TGA 8000. In a typical experiment, ca. 1–3 mg polymer material and one 3 mm grinding sphere were loaded into a crucible equipped with a round bottom quartz inlay to allow for better polymer–catalyst contact, and heated from 50 °C to 600 °C using a heating rate of 10 °C min^{-1} under a N_2 flow of 45 ml min^{-1}. Shown weight loss curves are min–max normalized.

Thermogravimetric analysis–mass spectrometry (TGA-MS) was performed on a Perkin Elmer TGA 8000 coupled to a Hiden Analytical MS system. In a typical experiment, ca. 1–2 mg sulfated zirconia powder was loaded into a crucible and heated from 30 °C to 1100 °C with a

heating rate of 10 °C min^{-1} under an Ar flow of 25 ml min^{-1}. During heating, the MS recorded m/z values of 18, 32, 40, 48, 64, and 80. Shown weight loss curves are max normalized.

Gel permeation chromatography (GPC) was performed on a Polymer Char (Valencia, Spain) GPC-IR instrument equipped with an infrared detector (IR4), three PLgel Olexis (300 × 7.5 mm, Agilent Technologies) columns in series and a PLGel Olexis (50 × 7.5 mm, Agilent Technologies) guard column. 1,2,4-Trichlorobenzene containing butylated hydroxytoluene (300 ppm) was used as eluent at a flow rate of 1.0 ml min^{-1} with a column temperature of 150 °C. The PP samples were prepared with a concentration of 1 mg ml^{-1} with heptane as an internal standard. The samples were dissolved at 160 °C under nitrogen for 1 h under continuous gentle shaking and filtered prior to injection. The molecular weight was calculated with respect to polystyrene standards (Polymer Laboratories, M_n = 5,310 up to M_n = 1,510,000 g mol^{-1}) and converted to polypropylene equivalents.

Scanning electron microscopy (SEM) with energy-dispersive X-ray (EDX) spectroscopic analysis was performed on a Thermo Scientific Phenom ProX microscope. The grinding spheres were pressed onto a double-sided adhesive and conductive carbon tape, which was attached to an aluminum SEM stub. SEM images were acquired using an acceleration potential of 10 kV. EDX elemental maps were obtained at 15 kV, with an imaging time of 50 ms per pixel and a resolution of 64 by 64 pixels. The shown percentages for elemental composition were determined by EDX spectroscopy and normalized to 100% for the shown elements. For better visibility, shown EDX elemental maps were modified in terms of hue and saturation in a consistent way for each element across the whole image.

Raman microscopy was performed on a Renishaw inVia microscope equipped with a 532 nm laser at a laser power of 5%. The spectra region of interest was between 100 and 1800 cm^{-1}. The spectrum at a certain spot was typically acquired using 10 scans with a measurement time of 20 s each, leading to a total acquisition time of 200 s.

Differential scanning calorimetry (DSC) was performed on a Mettler Toledo DSC 3 STARe instrument. Ca. 10 mg polymer were loaded into aluminum pans and heated under nitrogen atmosphere according to the following program: 5 min at 20 °C, then −10 °C min^{-1} to −50 °C, 5 min at −50 °C min^{-1}, then 10 °C min^{-1} to 250 °C, then 5 min at 250 °C, then −10 °C min^{-1} to −50 °C, then 5 min at −50 °C min^{-1}, then 10 °C min^{-1} to 250 °C. For the determination of degrees of crystallinity, the obtained curves were corrected with a linear baseline, integrated between 100 and 180 °C for melting and between 70 and 150 °C for crystallization with a trapezoidal method, and divided by 190 J g^{-1} as the heat of fusion for PP.

Gas chromatography (GC) with FID was performed on a Thermo Scientific Trace 1300 instrument. To prepare the analysis, 1 ml dichloromethane (DCM, Thermo Scientific, 99.8%) was added to the milling container directly after milling. The gas inlet and outlet were closed with caps and the container was shaken for 1 min at 10 Hz. The liquid phase was removed and filtered with a syringe filter, evaporated to dryness in air, after which 0.1 ml DCM and butyl decanoate (Aldrich, 98%) as an internal standard were added and the resulting solution was analyzed.

Density functional theory (DFT) calculations[1,2] were performed with the Vienna ab initio Simulation Package (VASP.6.2.1).[3,4] The generalized gradient approximation (GGA) with Perdew–Burke–Ernzerhof (PBE) exchange and correlation functional was used to account for the exchange–correlation energy.[5] The electron–ion interactions were described using the projected augmented wave (PAW) method and the plane-wave (PW) basis set.[3,4] The kinetic energy cut-off of the plane wave basis set was set to 520 eV. The convergence criterion for energy calculation and structure relaxation was set to a self-consistent field (SCF) threshold of 10^{-5} eV and a

maximum force threshold of 0.05 eV/Å. The bulk structures for tetragonal (mp-2574: t-ZrO_2) and monoclinic (mp-2858: m-ZrO_2) phases of ZrO_2 were obtained from the Materials project database. The obtained bulk structures had lattice parameters (for t-ZrO_2: a=b=3.60 Å, c=5.23 Å; and for m-ZrO_2: a=5.15 Å, b=5.23 Å, c=5.33 Å) in agreement with the experiments (for t-ZrO_2: a=b=3.61 Å, c=5.18 Å; and for m-ZrO_2: a=5.15 Å, b=5.21 Å, c=5.32 Å).[6] The stable surfaces t-ZrO_2 (101) and m-ZrO_2 (−111) were cleaved from their corresponding bulk structures. The supercells of size 2 × 3 × 1 were used for both phases of ZrO_2. However, for t-ZrO_2 (101) with SO_4 group and O-vacancies, the supercell of size 2 × 6 × 1 was used. Γ-centered k-meshes with sizes of 2 × 2 × 1 and 2 × 1 × 2 were used for sampling the Brillouin zones in the cases (t-ZrO_2 (101)) and monoclinic (m-ZrO_2 (−111)) slab models of ZrO_2, respectively. For t-ZrO_2 (101) with SO_4 group and O-vacancies, a Γ-centered k-mesh of size 2 × 1 × 1 was used. For all cases of t-ZrO_2 (101), the slabs were composed of three stoichiometric layers of t-ZrO_2, where the bottom two stoichiometric layers were fixed to mimic the bulk. For all cases of m-ZrO_2 (−111), the slabs were composed of four stoichiometric layers of m-ZrO_2, where the bottom two stoichiometric layers were fixed to mimic the bulk. Gaussian-type smearing with a width of 0.05 eV was applied for the electronic energy density of states. Vacuum distances of 15 Å and 10 Å were added along the z direction in the slab models of t-ZrO_2 (101) and m-ZrO_2 (−111), respectively, to minimize interaction with the periodic images, and then dipole corrections were applied in the vacuum (z) direction. The van der Waals interactions were described by the DFT-D3BJ method developed by Grimme *et al.*.[7,8] The adsorption energies E_{ads} were calculated according to **Eq. 4**, where $E_{\mathrm{slab+reactant}}$ is the total energy of the slab with a reactant adsorbed on it, E_{slab} is the total energy of the clean slab, and E_{reactant} is the total energy of the reactant.

$$E_{\mathrm{ads}} = E_{\mathrm{slab+reactant}} - E_{\mathrm{slab}} - E_{\mathrm{reactant}} \quad (4)$$

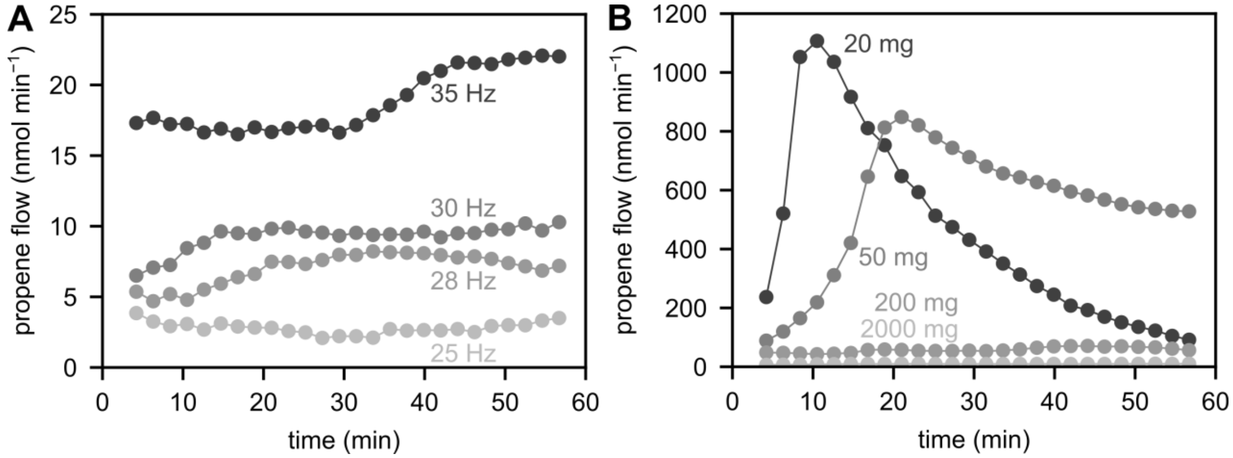

Figure S1. Propene flow during milling of model PP with untreated ZrO_2 grinding spheres. **(A)** Milling of 2 g model PP at 25, 28, 30, and 35 Hz. **(B)** Milling of 20, 50, 200, and 2000 mg model PP at 30 Hz. Propene flows at lower filling degrees are much higher due to the more direct force transfer between grinding spheres and plastic, and the mitigation of the "cushioning effect".

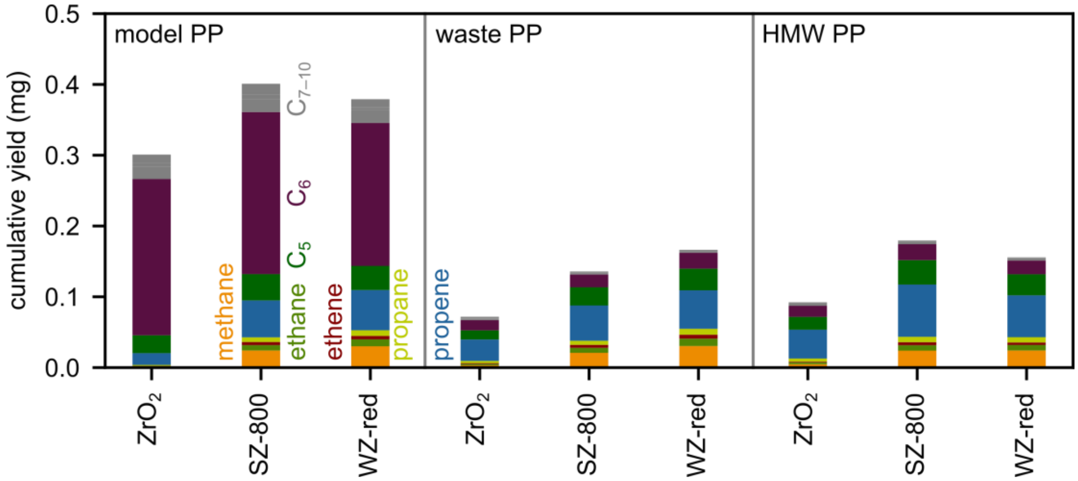

Figure S2. Cumulative yields obtained during milling of 2 g PP for 1 h at 30 Hz with sulfated (SZ-800), tungstated (WZ-red), and untreated (ZrO_2) grinding spheres.

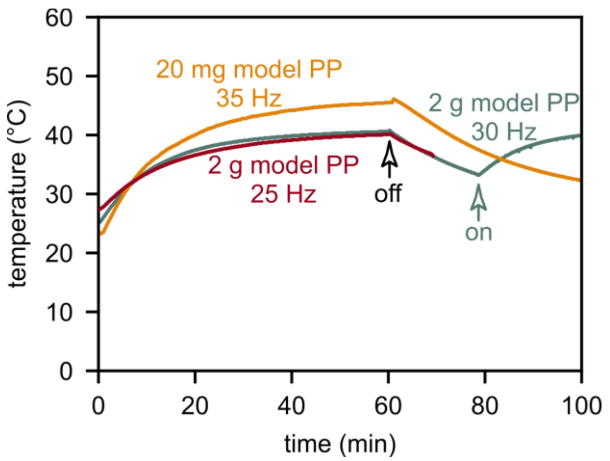

Figure S3. Temperature profile during milling of 2 g model PP at 25 Hz, 2 g model PP at 30 Hz, and 20 mg model PP at 35 Hz. The agitation was turned off and on at the indicated points. The temperature was measured on the surface of the grinding container using an attached thermocouple.

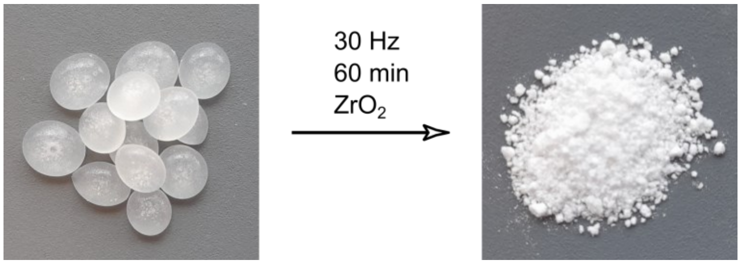

Figure S4. Photographs of model PP before and after milling for 60 min at 30 Hz with untreated ZrO_2 spheres.

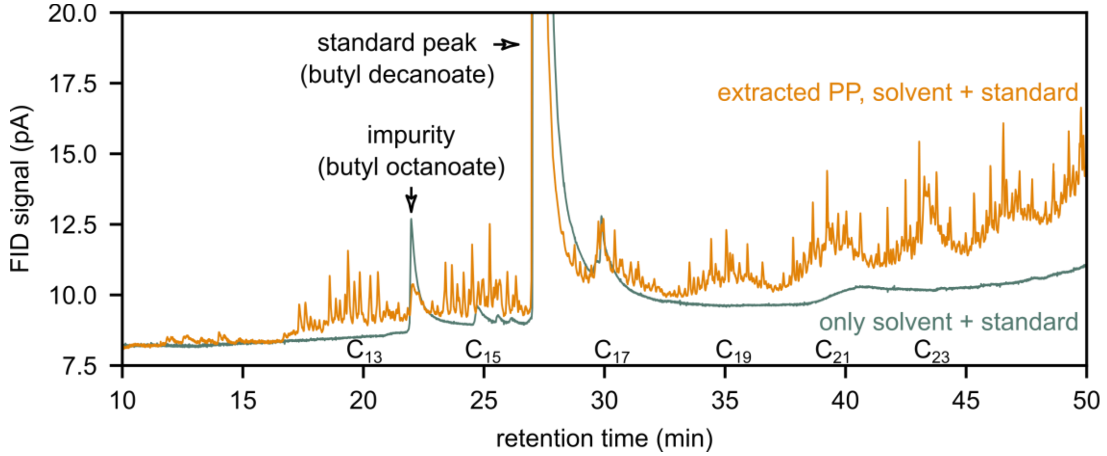

Figure S5. Gas chromatogram after extraction of milled residue. 20 mg model PP were milled for 1 h at 30 Hz and extracted with dichloromethane. Butyl decanoate was used as a standard for quantification, and we estimate a total of ca. 0.7 mg residual hydrocarbons extracted. Cuthbertson *et al.* observed a similar pattern after solvent extraction of model PP and reported a "complex mixtures of wax and fatty acids" as additives.[9] We therefore believe that a significant fraction of our observed higher hydrocarbons are actually additives rather than direct reaction products.

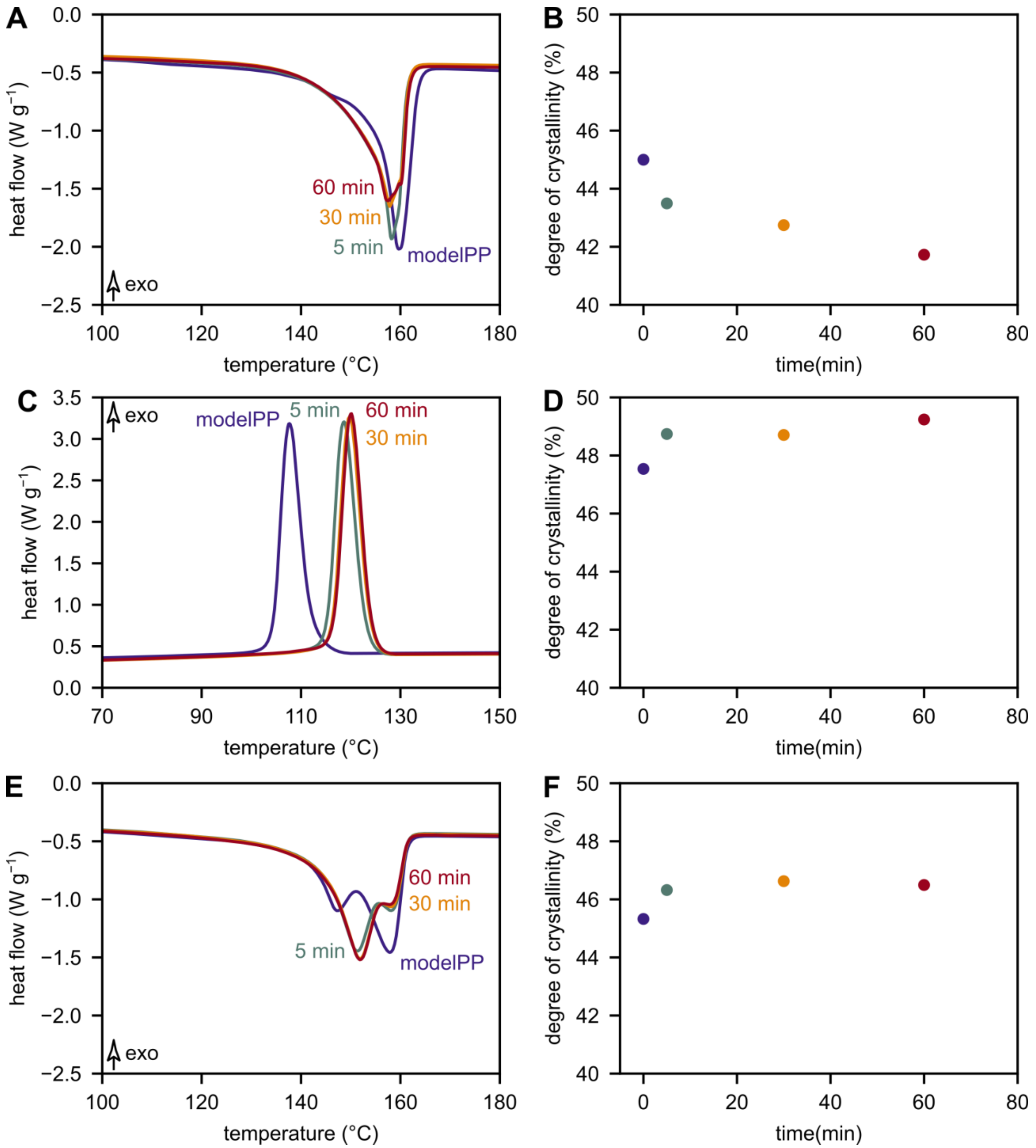

Figure S6. Differential scanning calorimetry data. **(A)** First melting peak and **(B)** degree of crystallinity as determined by integration of the first melting peak. **(C)** Crystallization peak and **(D)** degree of crystallinity as determined by integration of the crystallization peak. **(E)** Second melting peak and **(F)** degree of crystallinity as determined by integration of the second melting peak.

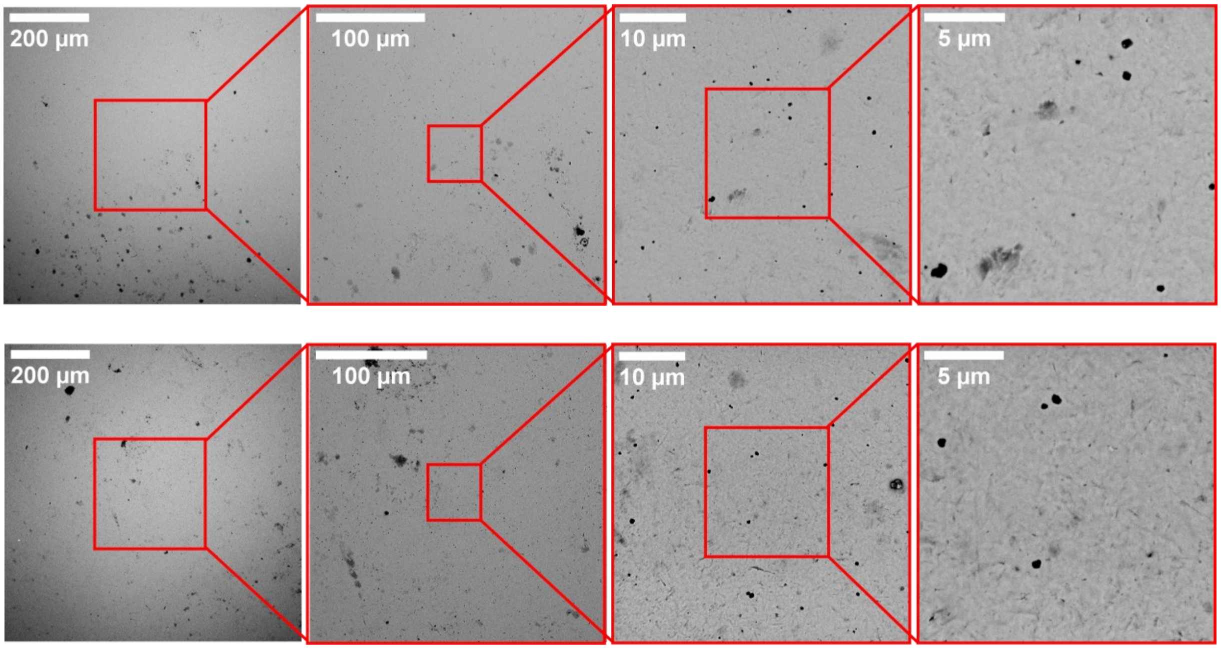

Figure S7. SEM images of untreated 3 mm zirconia grinding spheres.

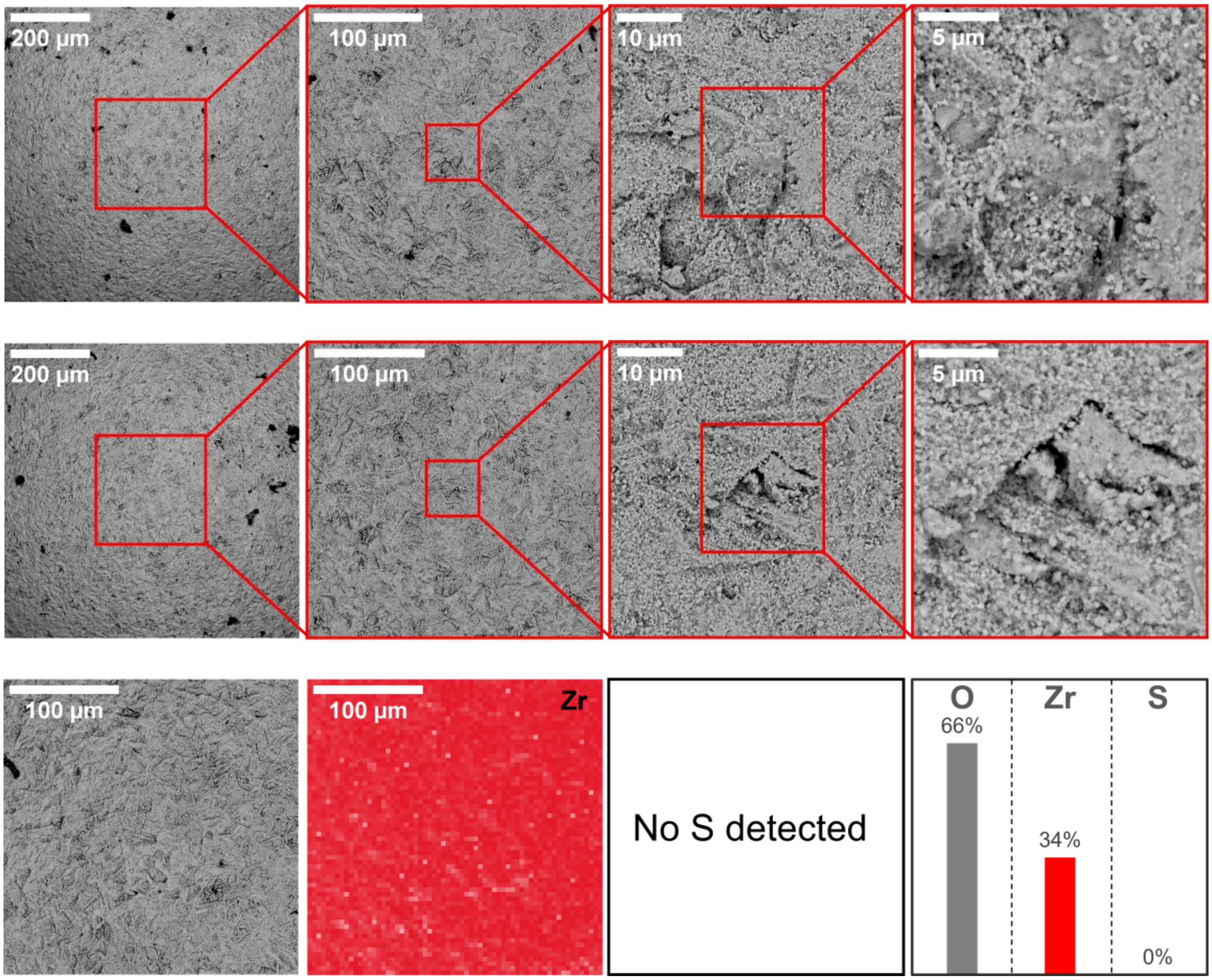

Figure S8. SEM images and energy-dispersive X-ray spectroscopy (EDX) mappings of 3 mm sulfated zirconia (SZ-800) grinding spheres.

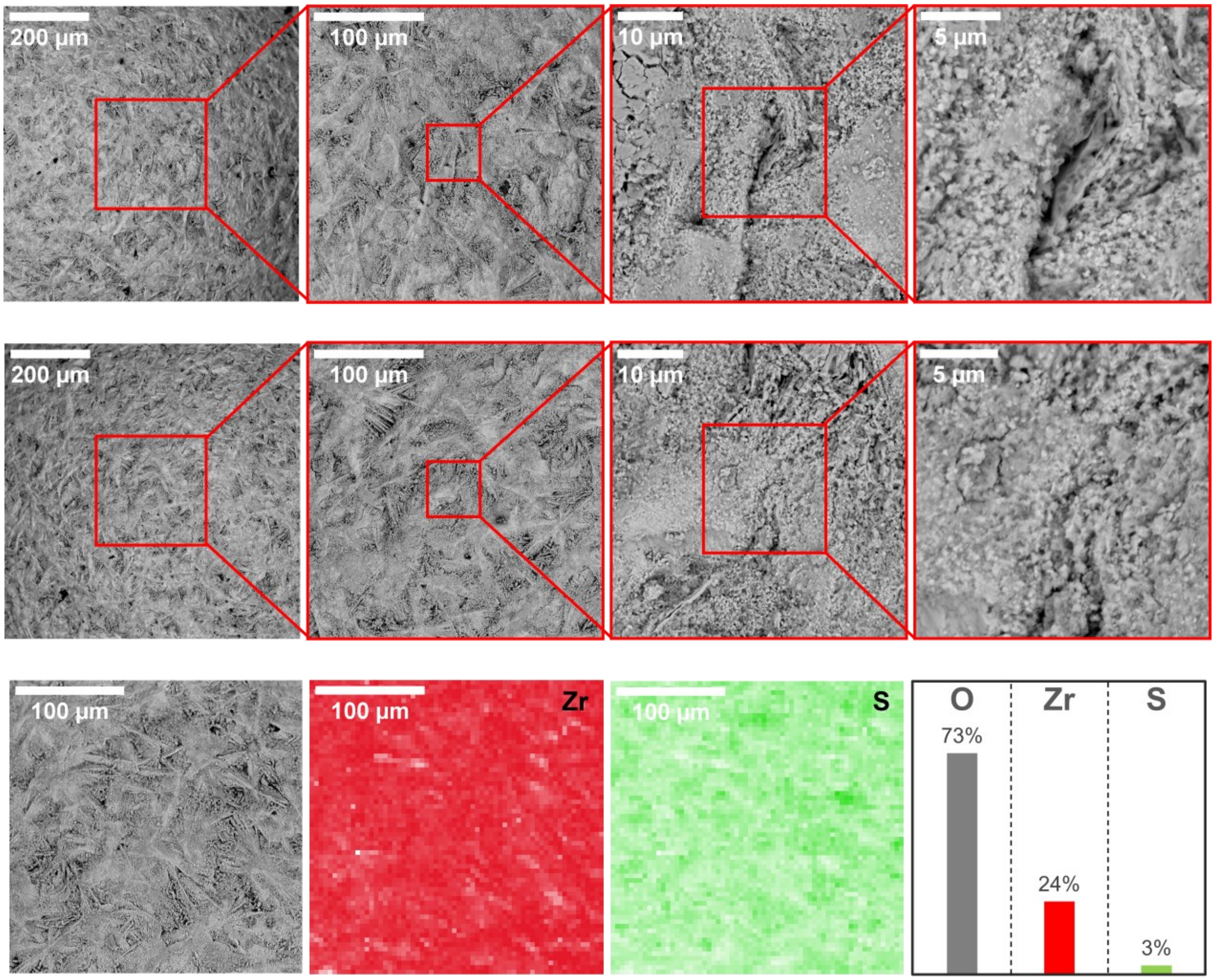

Figure S9. SEM images and EDX mappings of 3 mm sulfated zirconia (SZ-650) grinding spheres.

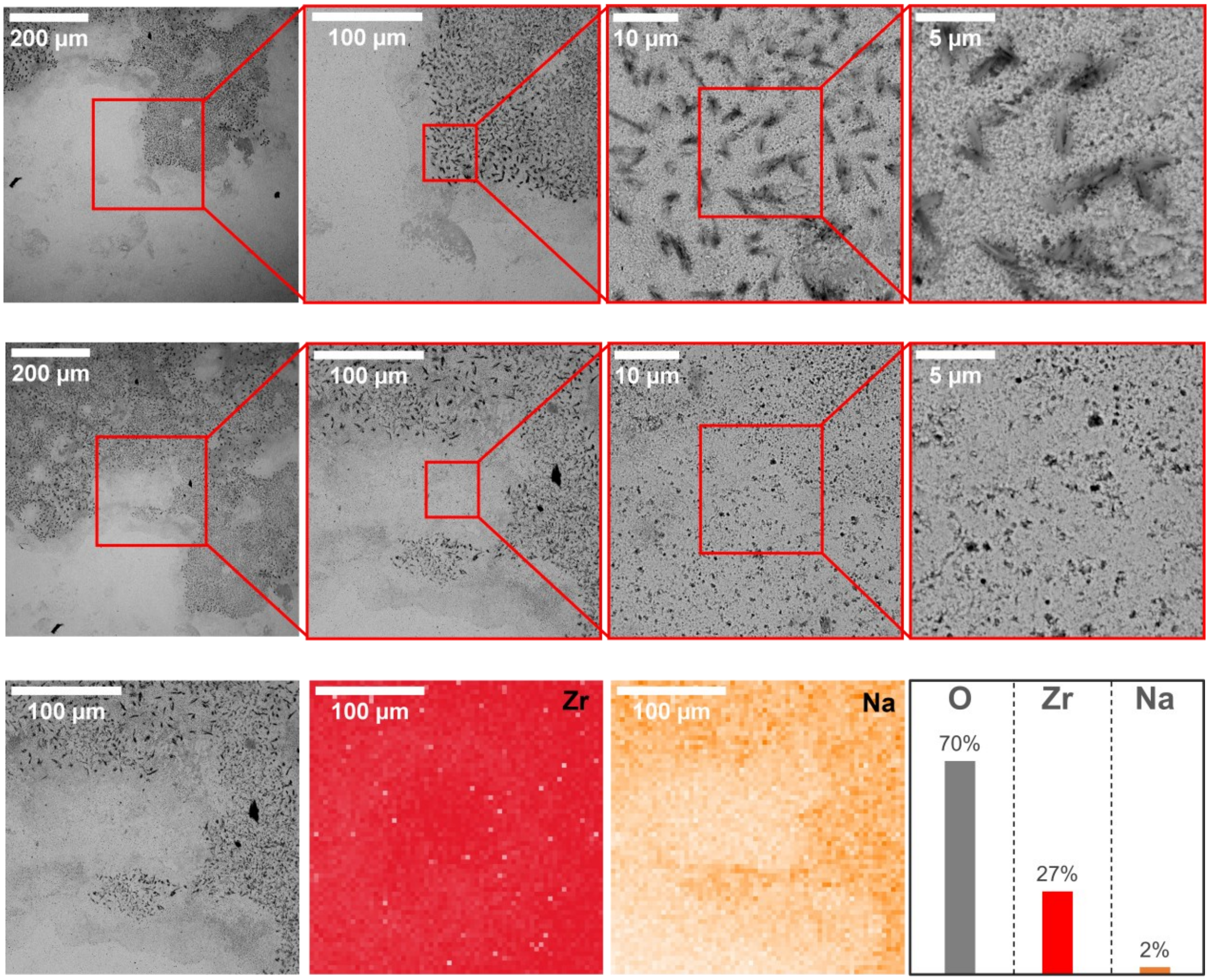

Figure S10. SEM images and EDX mappings of 3 mm NaOH-etched zirconia grinding spheres.

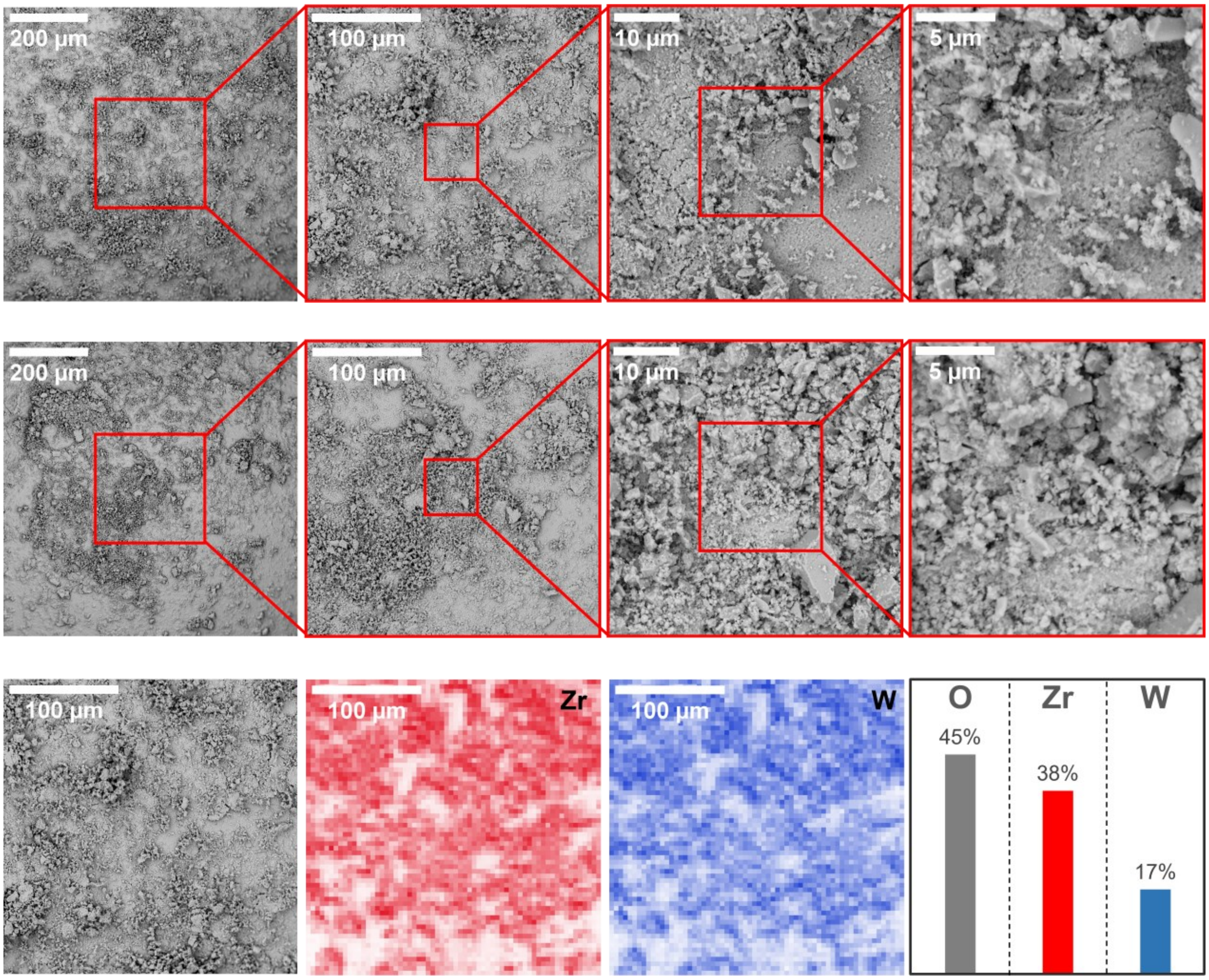

Figure S11. SEM images and EDX mappings of 3 mm tungstated zirconia grinding spheres after calcination, but before reduction (WZ-calc).

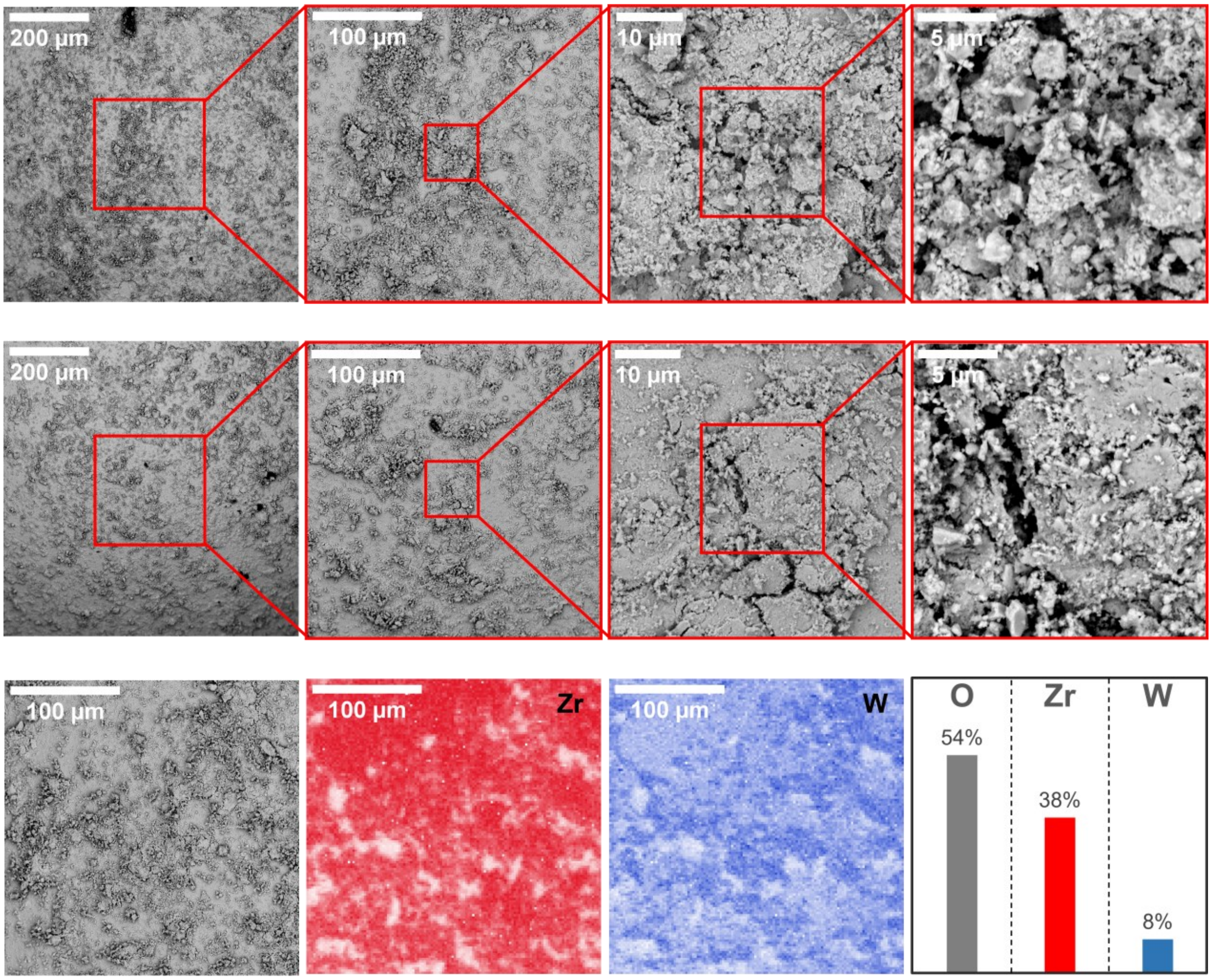

Figure S12. SEM images and EDX mappings of 3 mm tungstated zirconia grinding spheres after calcination and subsequent reduction (WZ-red).

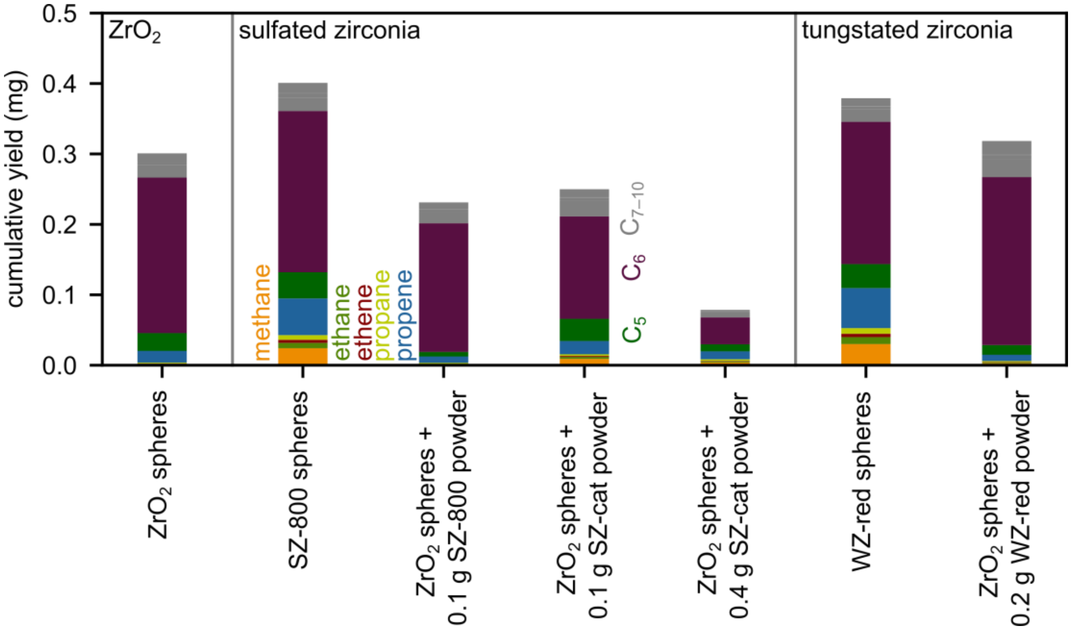

Figure S13. Cumulative yields obtained during milling of 2 g PP for 1 h at 30 Hz with sulfated (SZ-800), tungstated (WZ-red), and untreated (ZrO_2) grinding spheres, compared to milling with untreated ZrO_2 spheres and 0.1 g or 0.4 g synthesized SZ catalyst powder (SZ-cat), 0.1 g SZ-800 synthesis residue powder or 0.2 g WZ powder catalysts. We believe that a relevant portion of C_6 formed from model PP stems from additives added during plastic manufacturing, such as UV stabilizers or "complex mixtures of wax and fatty acids", as reported by Cuthbertson *et al*.[9] These can adsorb on the high surface area sulfated zirconia, especially at high loadings, which reduces the detection of C_6.

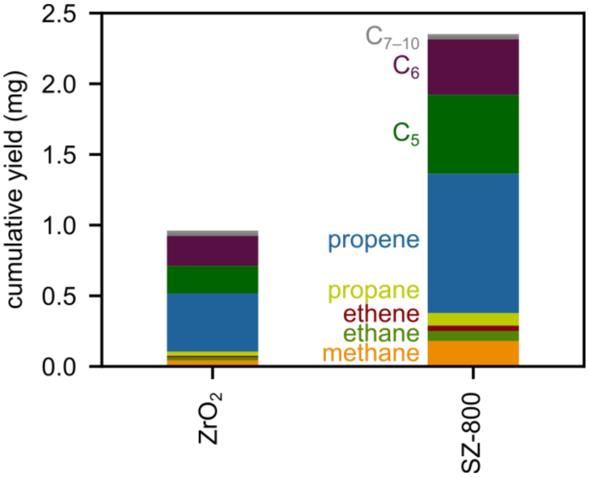

Figure S14. Cumulative yields obtained during milling of 100 mg PP for 1 h at 30 Hz with sulfated (SZ-800), and untreated (ZrO_2) grinding spheres.

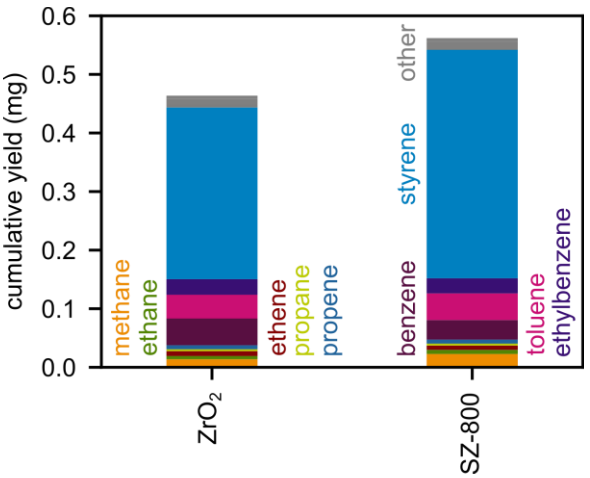

Figure S15. Cumulative hydrocarbon yields obtained via milling of 2 g PS for 1 h at 30 Hz with sulfated (SZ-800), and untreated (ZrO_2) grinding spheres.

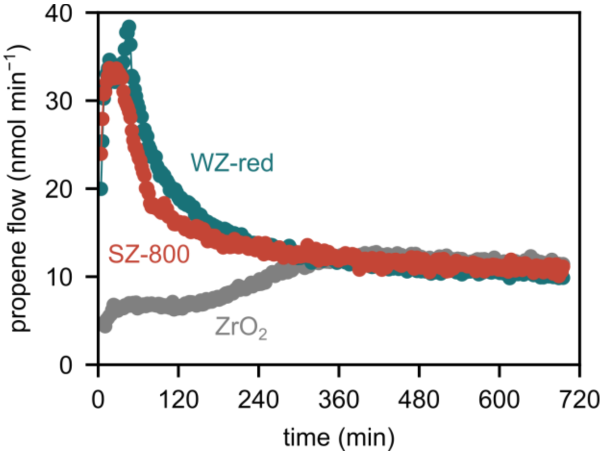

Figure S16. Propene flow during milling of model PP for 12 h at 30 Hz with sulfated (SZ-800), tungstated (WZ-red), and untreated (ZrO_2) grinding spheres.

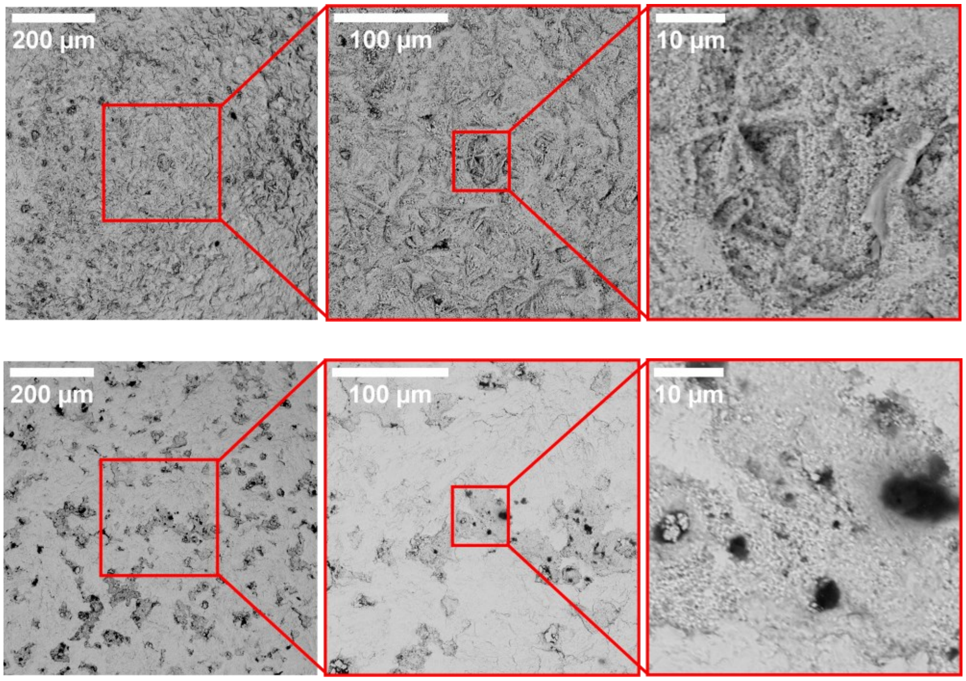

Figure S17. SEM images of a 10 mm SZ-800 grinding sphere before milling (top), and a 10 mm SZ-800 grinding sphere after 1 h of milling model PP at 30 Hz (bottom).

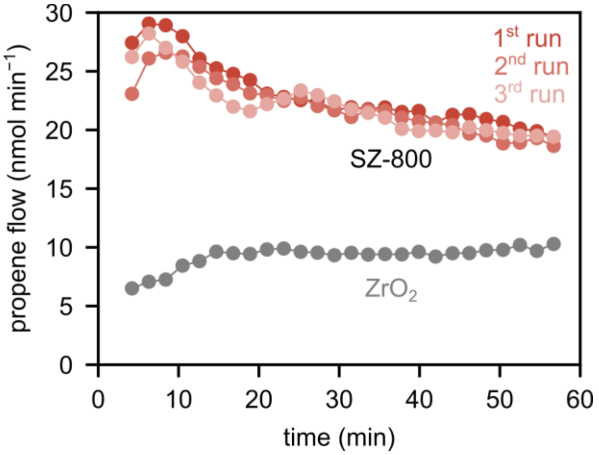

Figure S18. Propene flow during milling of model PP at 30 Hz with sulfated (SZ-800), and untreated (ZrO_2) grinding spheres. The sulfated spheres were separated from the plastic after each run, washed, and re-sulfated by subjecting them to the original synthesis conditions.

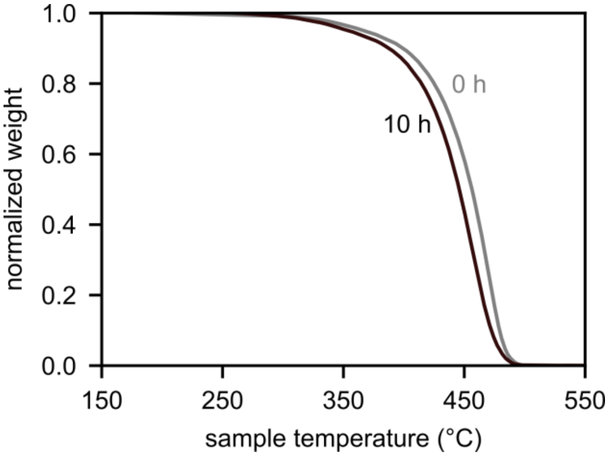

Figure S19. TGA profiles of model PP before and after milling with untreated ZrO_2 grinding spheres for 10 h. No increase in degradation temperature, which would indicate cross-linking, was observed.

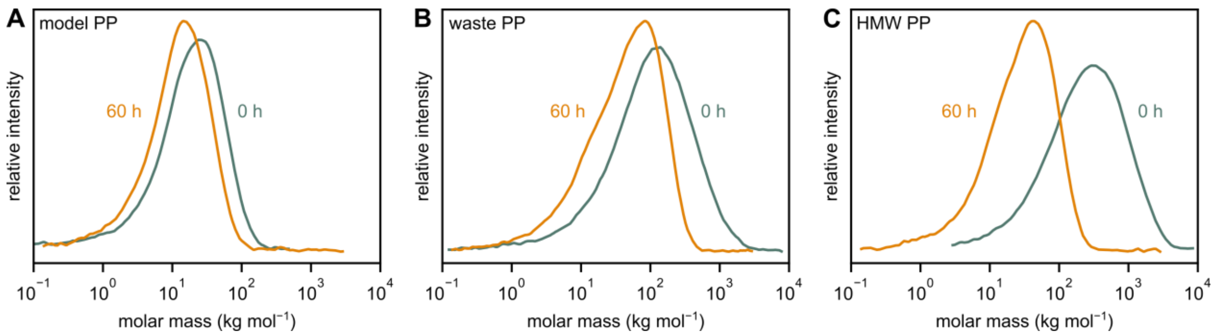

Figure S20. Molar mass distributions before and after milling **(A)** model PP, **(B)** waste PP, and **(C)** HMW PP for 60 h with untreated ZrO_2 spheres. The shift in molar mass is more pronounced for higher starting M_w polymers (**Table S2**), which illustrates preferred cleavage for longer chain under mechano-chemical activation.

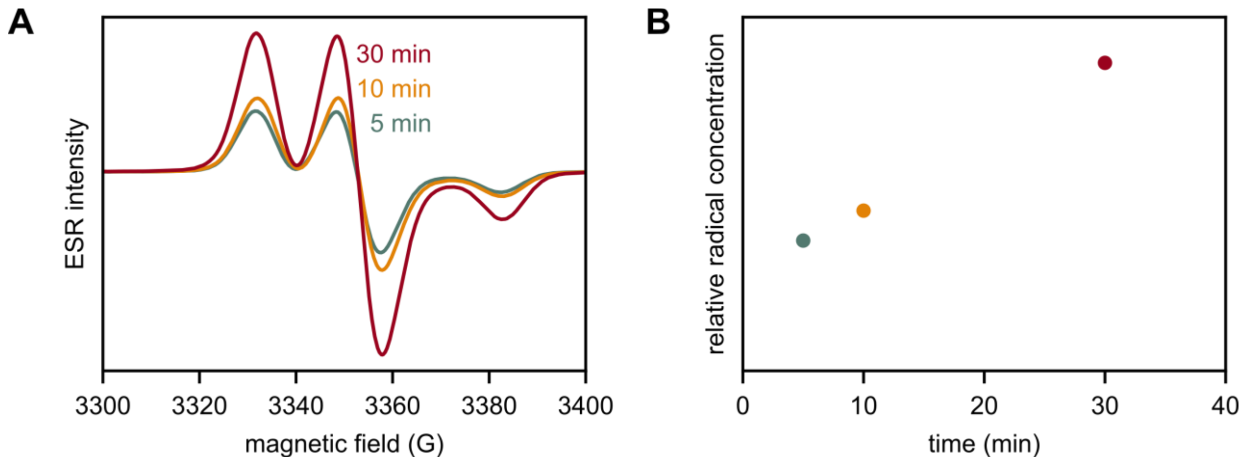

Figure S21. (A) Mass-normalized ESR spectra after milling model PP with 50 mg nitrosobenzene for 5, 10, and 30 min, and **(B)** the integrals of microwave absorption as a measure of trapped radical concentration against time.

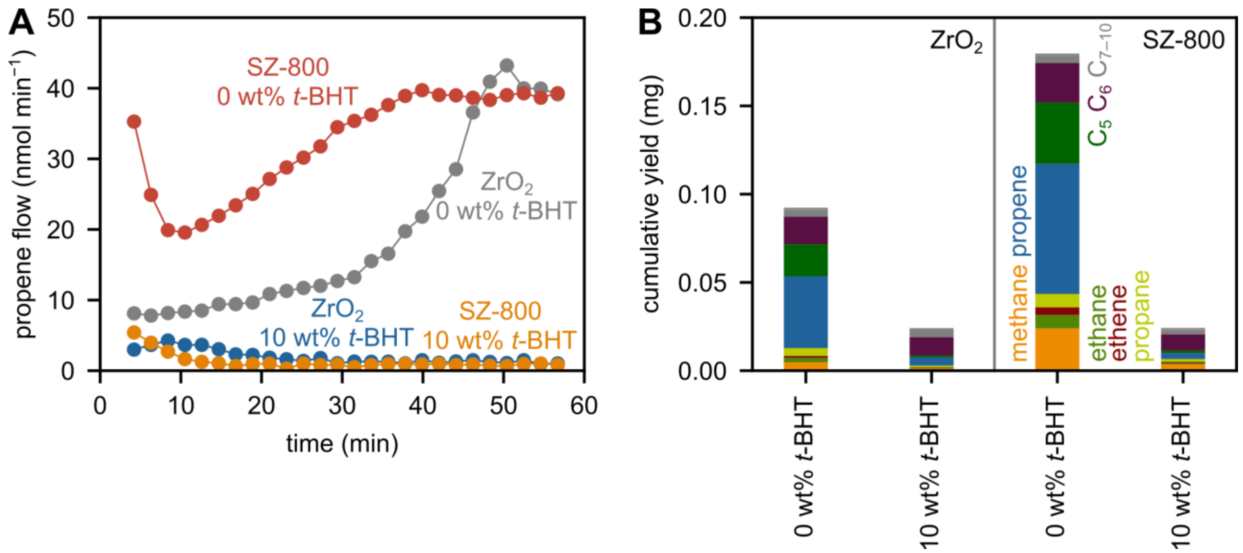

Figure S22. Radical quenching experiments with *t*-BHT: **(A)** Quenching of propene formation with 10 wt% *t*-BHT while milling HMW PP with sulfated (SZ-800), and untreated (ZrO_2) spheres. **(B)** Cumulative hydrocarbon yields obtained via milling of 2 g HMW PP for1 h.

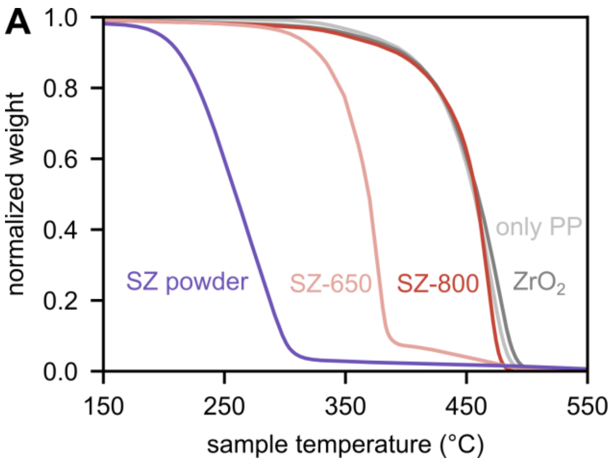

Figure S23. TGA profiles of model PP with untreated (ZrO_2) and **(A)** sulfated (SZ) grinding spheres and powder catalyst, and **(B)** tungstated (WZ) grinding spheres and powder catalyst, and a NaOH-etched ZrO_2 grinding sphere.

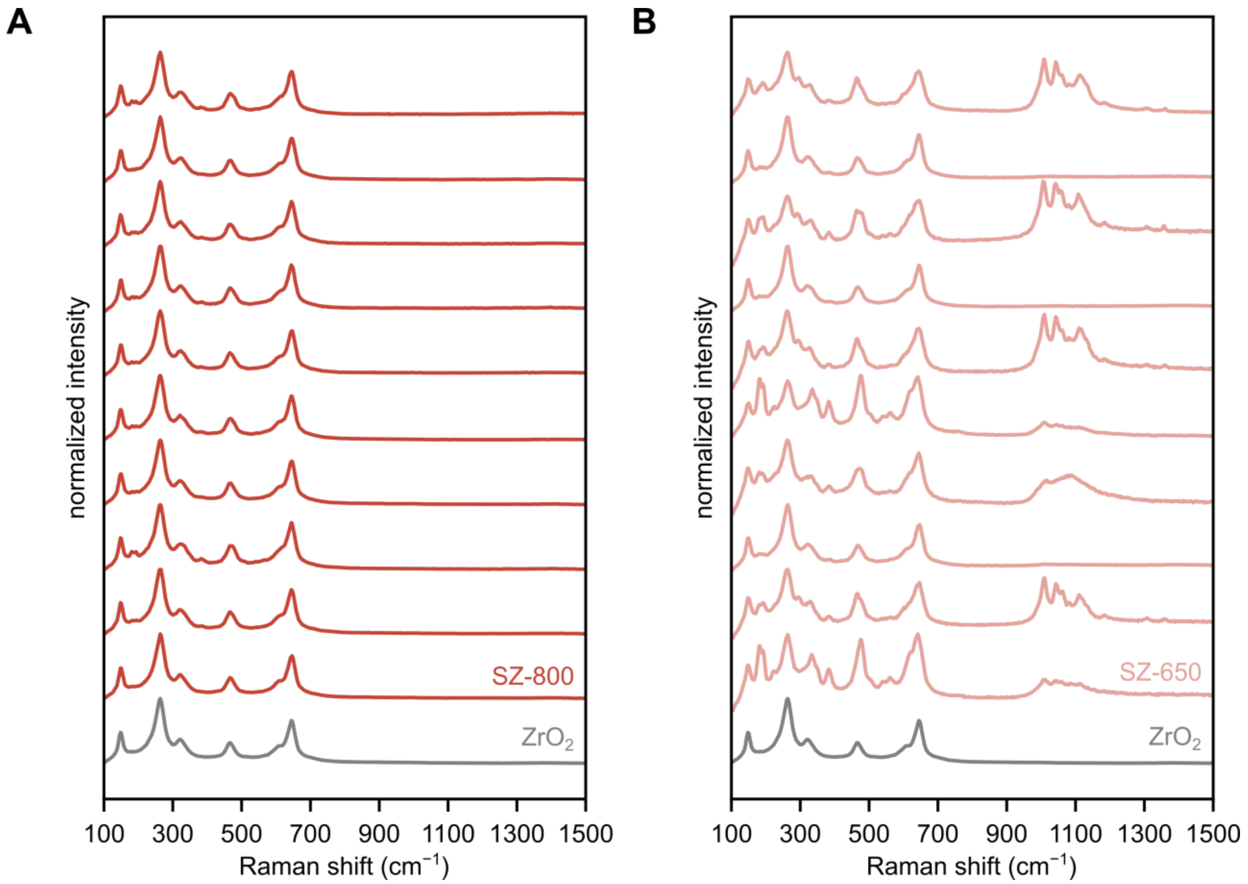

Figure S24. Raman spectra of untreated ZrO_2 grinding spheres and at 10 different spots of **(A)** sulfated (SZ-800) ZrO_2 grinding spheres and **(B)** sulfated (SZ-650) ZrO_2 grinding spheres.

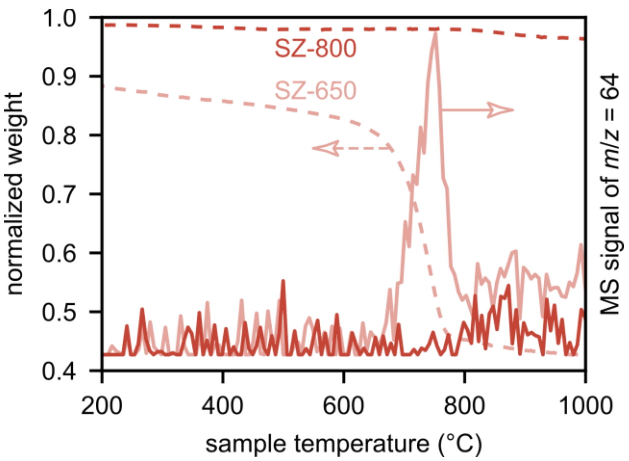

Figure S25. TGA–mass spectrometry measurements of SZ-650 and SZ-800 synthesis residues. Surface sulfate groups decompose into mainly SO_2 ($m/z = 64$) upon heating. Compared to SZ-800, SZ-650 shows a much higher weight loss, corresponding to a higher sulfate concentration.

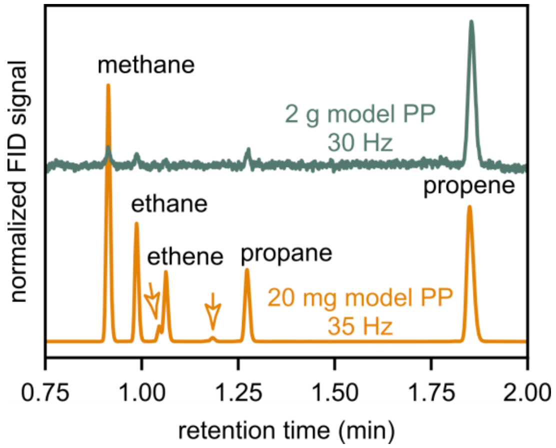

Figure S26. Representative normalized gas chromatograms recorded during milling of 2 g model PP at 30 Hz, and 20 mg model PP at 35 Hz. While the former case only features methane, ethane, ethene, propane, and propene, the latter shows additional species being formed.

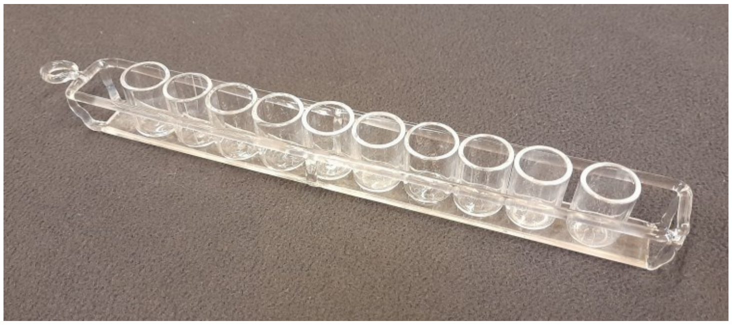

Figure S27. Cup holder and quartz cups used for the treatment of zirconia grinding spheres with sulfuric acid.

Table S1. Number average molar mass (M_n), weight average molar mass (M_w) and dispersity (M_w/M_n) of waste PP before and after milling for 10 and 60 h with untreated (ZrO_2) and sulfated (SZ-800) grinding spheres.

	milling time	M_n (g mol^{-1})	M_w (g mol^{-1})	M_w/M_n (–)
	0 h	17,700	209,700	11.85
ZrO_2	10 h	15,300	145,000	9.48
	60 h	11,700	68,300	5.82
SZ-800	10 h	15,000	132,400	8.84
	60 h	11,000	65,100	5.92

Table S2. Number average molar mass (M_n), weight average molar mass (M_w), dispersity (M_w/M_n), and decrease of M_w during milling of model, waste, and HMW PP for 60 h with untreated ZrO_2 spheres.

	milling time	M_n (g mol^{-1})	M_w (g mol^{-1})	M_w/M_n (–)	ΔM_w (%)
model PP	0 h	4,300	28,100	6.51	
	60 h	4,800	14,400	2.98	−49%
waste PP	0 h	17,700	209,700	11.85	
	60 h	11,700	68,300	5.82	−67%
HMW PP	0 h	83,600	456,900	5.46	
	60 h	10,600	39,900	3.76	−91%

Table S3. Comparison of ESR spectroscopic *g* values, as derived from ESR for ZrO_2 in this work, and the comparison with recent work of Gionco *et al.*[10]

Assignment		$g_x = g_y = g_\perp$	$g_z = g_\parallel$
Zr^{3+}	this work	1.977	1.958
	Gionco *et al.*	1.9768	1.9589
F-center (e^-)	this work	2.003	
	Gionco *et al.*	2.0024	

Table S4. Adsorption energies of secondary carbon-centered radicals by DFT calculations on tetragonal and monoclinic ZrO_2 surfaces containing sulfate groups (SO_4), Zr^{3+} and oxygen vacancies (Zr^{3+}–V_O), a combination of SO_4 and Zr^{3+}–V_O, Zr^{3+}–O–W^{5+} surface species, S–OH groups, and Zr–OH groups.

surface species	E_{ads} / eV	
	tetragonal ZrO_2	monoclinic ZrO_2
pristine	−0.65	−0.51
SO_4	−0.56	−0.25
Zr^{3+}–V_O	−2.72	−2.52
SO_4 and Zr^{3+}–V_O	−2.49	−2.73
Zr^{3+}–O–W^{5+}	−1.99	−2.43
S–OH[a]	−3.58	
Zr–OH[a]	−2.69	

[a] Reaction with the carbon-centered radical $R\cdot$ leads to hydrogen abstraction and R–H not bound to the surface.

Table S5. Clean slab models created from the *t*-ZrO_2 (101) bulk structure. Oxygen vacancies are marked with a black star.

Clean slab models	Top view	Side view
t-ZrO_2 (101) pristine	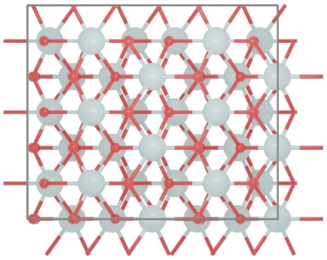	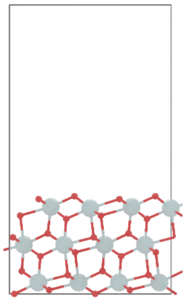
t-ZrO_2 (101) with O-vacancy	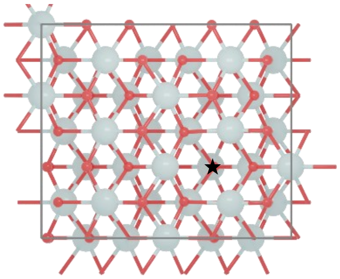	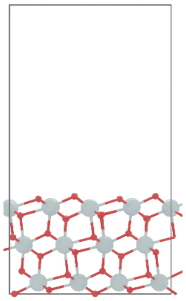
t-ZrO_2 (101) with SO_4 group	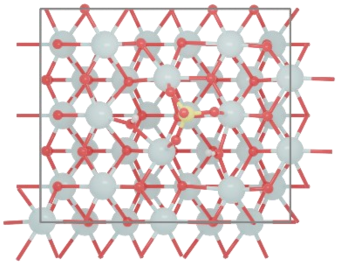	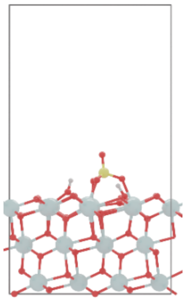
t-ZrO_2 (101) with SO_4 group & O-vacancy	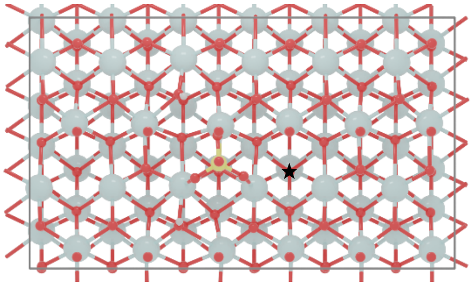	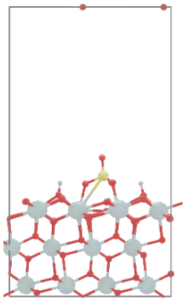
t-ZrO_2 (101) doped with W	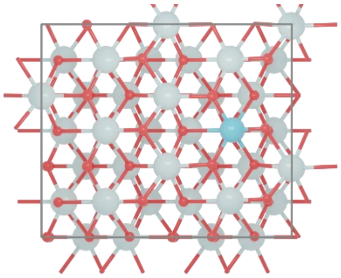	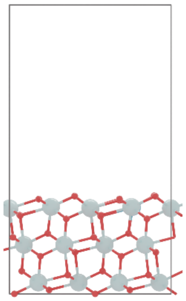

Table S6. Slab models created from the *t*-ZrO_2 (101) bulk structure and adsorption energies (E_{ads}) of the adsorbates (secondary carbon-centered radicals).

Slab models	Top view	Side view
t-ZrO_2 (101)<br>pristine<br>E_{ads} = −0.65 eV	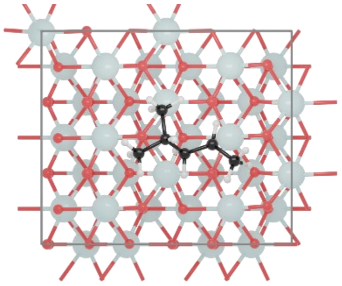	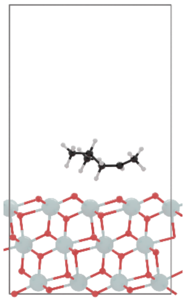
t-ZrO_2 (101)<br>with O-vacancy<br>E_{ads} = −2.72 eV	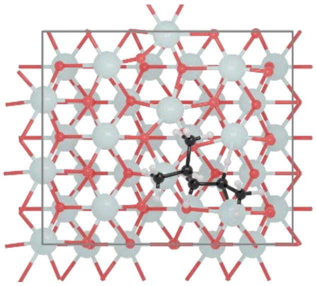	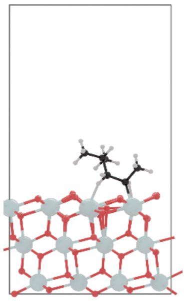
t-ZrO_2 (101)<br>with SO_4 group<br>E_{ads} = −0.56 eV	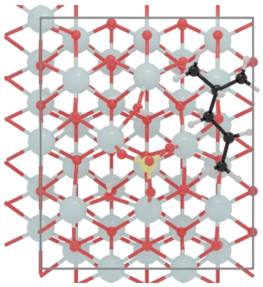	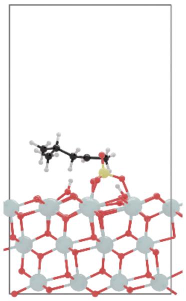
t-ZrO_2 (101)<br>with SO_4 group &<br>O-vacancy<br>E_{ads} = −2.49 eV	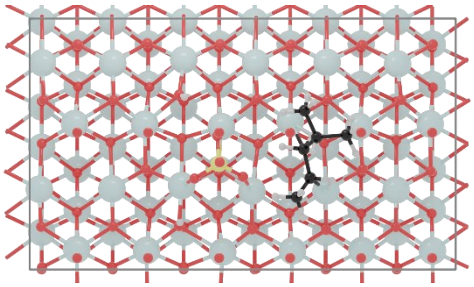	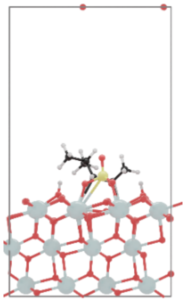
t-ZrO_2 (101)<br>doped with W<br>E_{ads} = −1.99 eV	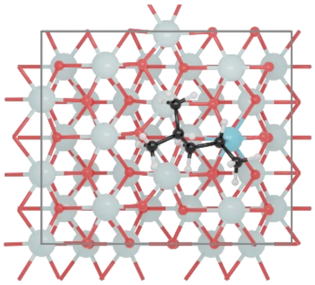	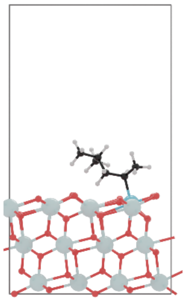

Table S7. Clean slab models created from the *m*-ZrO_2 (−111) bulk structure. Oxygen vacancies are marked with a black star.

Clean slab models	Top view	Side view
m-ZrO_2 (−111) pristine	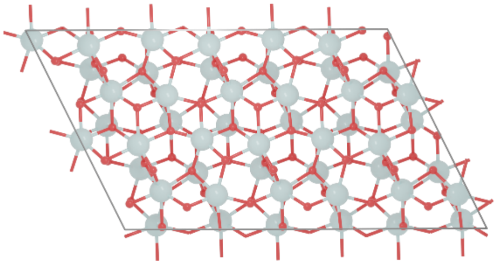	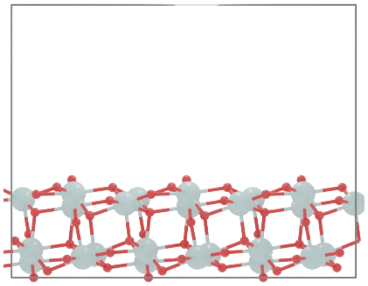
m-ZrO_2 (−111) with O-vacancy	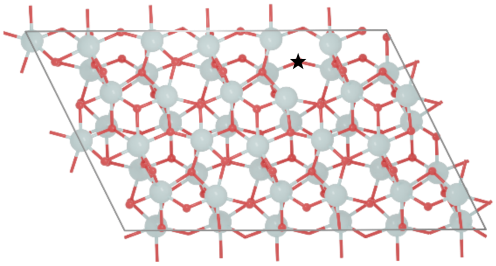	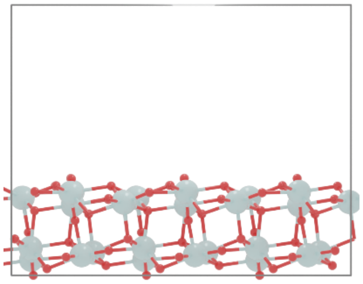
m-ZrO_2 (−111) with SO_4 group	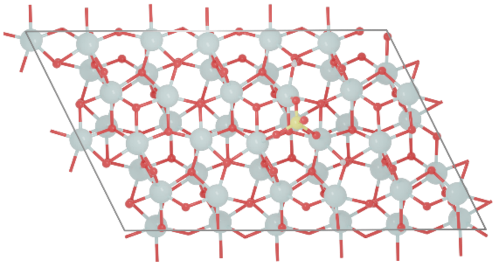	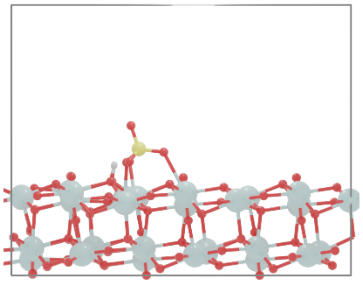
m-ZrO_2 (−111) with SO_4 group & O-vacancy	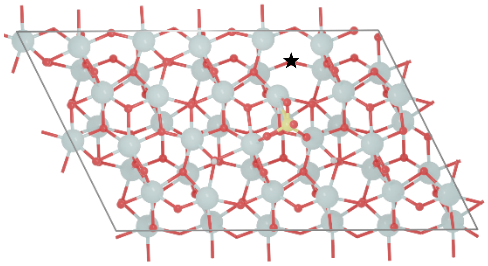	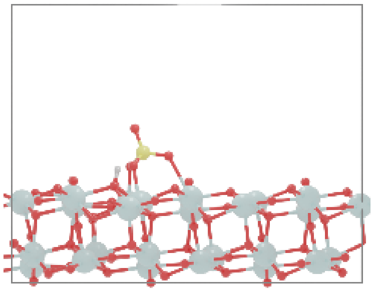
m-ZrO_2 (−111) doped with W	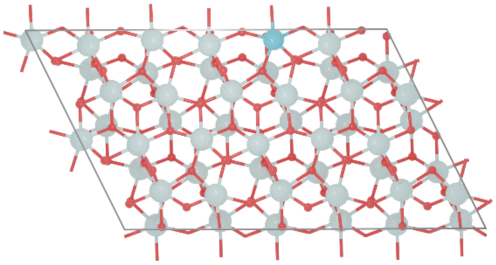	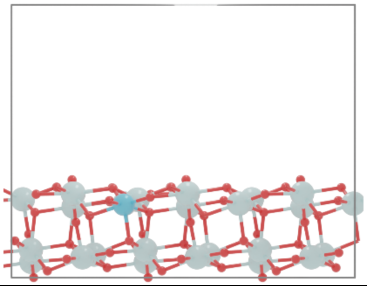

Table S8. Slab models created from the *m*-ZrO_2 (−111) bulk structure and adsorption energies (E_{ads}) of the adsorbates (secondary carbon-centered radicals).

Slab models	Top view	Side view
m-ZrO_2 (−111)<br>pristine<br>E_{ads} = −0.51 eV	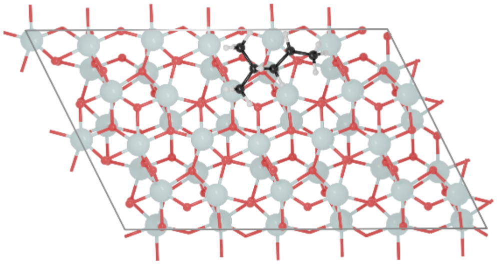	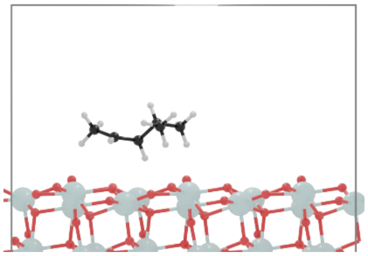
m-ZrO_2 (−111)<br>with O-vacancy<br>E_{ads} = −2.52 eV	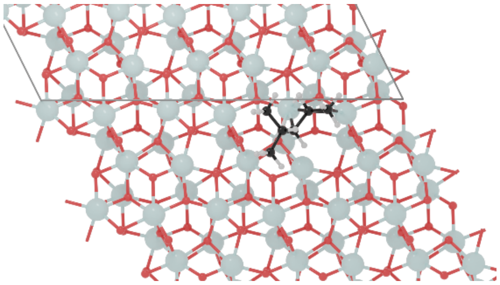	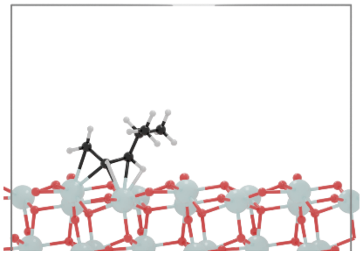
m-ZrO_2 (−111)<br>with SO_4 group<br>E_{ads} = −0.25 eV	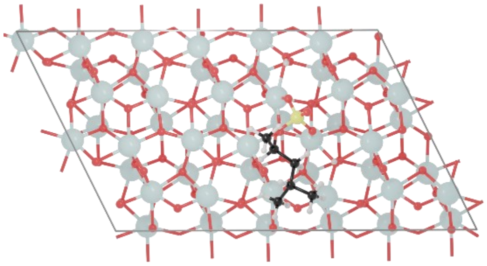	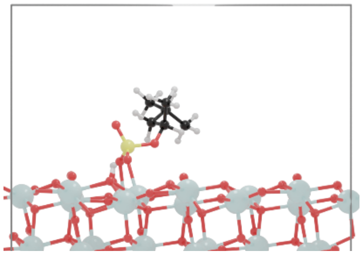
m-ZrO_2 (−111)<br>with SO_4 group &<br>O-vacancy<br>E_{ads} = −2.73 eV	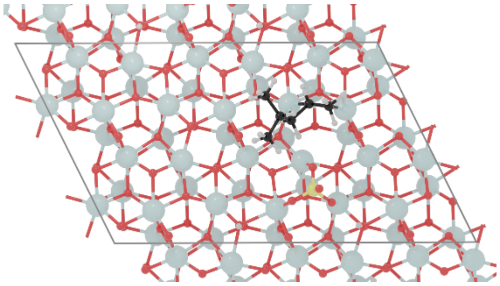	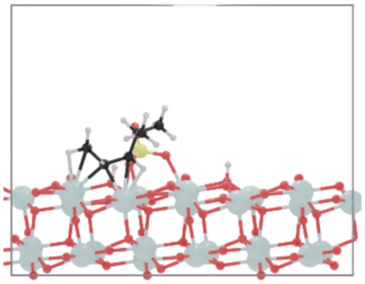
m-ZrO_2 (−111)<br>doped with W<br>E_{ads} = −2.43 eV	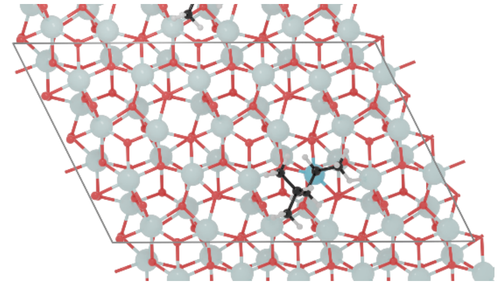	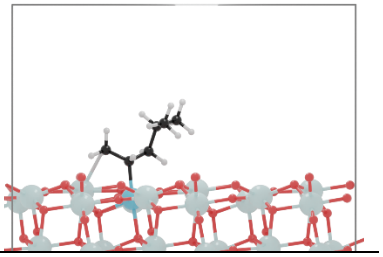

Table S9. Used polymer materials, shapes, and molar masses.

Denoted as	Source	Product	Shape	M_n (g mol^{-1})	M_w (g mol^{-1})
model PP	Sigma-Aldrich	428116	pellets	4,300	28,100
HMW PP	Ducor	DuPure G72TF	powder	83,600	456,900
waste PP	Albert Heijn	Blueberry bucket[a]	flakes	17,700	209,700
PE	Avient	UHMWPE	powder	31,800	390,900
PS	Sigma-Aldrich	430102	pellets	192,000[b]	
industrial waste		multilayer foils	flakes		

[a]Cleaned with deionized water prior to usage. [b]Supplier value.

References

(1) Hohenberg, P.; Kohn, W. Inhomogeneous Electron Gas. *Phys. Rev.* **1964**, *136* (3B), B864–B871. https://doi.org/10.1103/PhysRev.136.B864.

(2) Kohn, W.; Sham, L. J. Self-Consistent Equations Including Exchange and Correlation Effects. *Phys. Rev.* **1965**, *140* (4A), A1133–A1138. https://doi.org/10.1103/PhysRev.140.A1133.

(3) Kresse, G.; Furthmüller, J. Efficiency of Ab-Initio Total Energy Calculations for Metals and Semiconductors Using a Plane-Wave Basis Set. *Comput. Mater. Sci.* **1996**, *6* (1), 15–50. https://doi.org/10.1016/0927-0256(96)00008-0.

(4) Kresse, G.; Furthmüller, J. Efficient Iterative Schemes for Ab Initio Total-Energy Calculations Using a Plane-Wave Basis Set. *Phys. Rev. B* **1996**, *54* (16), 11169–11186. https://doi.org/10.1103/PhysRevB.54.11169.

(5) Perdew, J. P.; Burke, K.; Ernzerhof, M. Generalized Gradient Approximation Made Simple. *Phys. Rev. Lett.* **1996**, *77* (18), 3865–3868. https://doi.org/10.1103/PhysRevLett.77.3865.

(6) Howard, C. J.; Hill, R. J.; Reichert, B. E. Structures of ZrO 2 Polymorphs at Room Temperature by High-Resolution Neutron Powder Diffraction. *Acta Crystallogr. Sect. B Struct. Sci.* **1988**, *44* (2), 116–120. https://doi.org/10.1107/S0108768187010279.

(7) Grimme, S.; Antony, J.; Ehrlich, S.; Krieg, H. A Consistent and Accurate Ab Initio Parametrization of Density Functional Dispersion Correction (DFT-D) for the 94 Elements H-Pu. *J. Chem. Phys.* **2010**, *132* (15), 154104. https://doi.org/10.1063/1.3382344.

(8) Grimme, S.; Ehrlich, S.; Goerigk, L. Effect of the Damping Function in Dispersion Corrected Density Functional Theory. *J. Comput. Chem.* **2011**, *32* (7), 1456–1465. https://doi.org/10.1002/jcc.21759.

(9) Cuthbertson, A. A.; Lincoln, C.; Miscall, J.; Stanley, L. M.; Maurya, A. K.; Asundi, A. S.; Tassone, C. J.; Rorrer, N. A.; Beckham, G. T. Characterization of Polymer Properties and Identification of Additives in Commercially Available Research Plastics. *Green Chem.* **2024**, *26* (12), 7067–7090. https://doi.org/10.1039/D4GC00659C.

(10) Gionco, C.; Paganini, M. C.; Giamello, E.; Burgess, R.; Di Valentin, C.; Pacchioni, G. Paramagnetic Defects in Polycrystalline Zirconia: An EPR and DFT Study. *Chem. Mater.* **2013**, *25* (11), 2243–2253. https://doi.org/10.1021/cm400728j.
